# Phytochemical and biological assessment of secondary metabolites isolated from a rhizosphere strain, *Sphingomonas sanguinis* DM of *Datura metel*

**DOI:** 10.1186/s12906-024-04482-6

**Published:** 2024-05-25

**Authors:** Mohamed A. Awad, Sherif F. Hammad, Samir F. El-Mashtoly, Bahig El-Deeb, Hesham S. M. Soliman

**Affiliations:** 1 Biotechnology Program, Institute of Basic and Applied Science, Egypt-Japan University of Science and Technology (E-JUST), New Borg El-Arab City, Alexandria, 21934 Egypt; 2 Botany and Microbiology Department, Faculty of Science, Sohag University, Sohag, 82524 Egypt; 3 Department of Pharmaceutical Chemistry, Faculty of Pharmacy, Helwan University, Helwan, Cairo, Egypt; 4 Department of Pharmacognosy, Faculty of Pharmacy, Helwan University, Helwan, Cairo, Egypt; 5 PharmD Program, Egypt-Japan University of Science and Technology (E-JUST), New Borg El-Arab City, Alexandria, 21934 Egypt

**Keywords:** *Sphingomonas sanguinis* DM, Rhizosphere bacteria, *Datura metel*, Antimicrobial, Cytotoxicity

## Abstract

**Background:**

The plant roots excrete a large number of organic compounds into the soil. The rhizosphere, a thin soil zone around the roots, is a hotspot for microbial activity, making it a crucial component of the soil ecosystem. Secondary metabolites produced by rhizospheric *Sphingomonas sanguinis* DM have sparked significant curiosity in investigating their possible biological impacts.

**Methods:**

A bacterial strain has been isolated from the rhizosphere of *Datura metel*. The bacterium’s identification, fermentation, and working up have been outlined. The ethyl acetate fraction of the propagated culture media of *Sphingomonas sanguinis* DM was fractioned and purified using various chromatographic techniques. The characterization of the isolated compounds was accomplished through the utilization of various spectroscopic techniques, such as UV, MS, 1D, and 2D-NMR. Furthermore, the evaluation of their antimicrobial activity was conducted using the agar well diffusion method, while cytotoxicity was assessed using the MTT test.

**Results:**

The extract from *Sphingomonas sanguinis* DM provided two distinct compounds: n-dibutyl phthalic acid (**1**) and Bis (2-methyl heptyl) phthalate (**2**) within its ethyl acetate fraction. Furthermore, the 16S rRNA gene sequence of *Sphingomonas sanguinis* DM has been registered under the NCBI GenBank database with the accession number PP422198. The bacterial extract exhibited its effect against gram-positive bacteria, inhibiting *Streptococcus mutans* (12.6 ± 0.6 mm) and *Staphylococcus aureus* (10.6 ± 0.6 mm) compared to standard antibiotics. Conversely, compound **1** showed a considerable effect against phytopathogenic fungi such as *Alternaria alternate* (56.3 ± 10.6 mm) and *Fusarium oxysporum* (21.3 ± 1.5 mm) with a MIC value of 17.5 µg/mL. However, it was slightly active against *Klebsiella pneumonia* (11.0 ± 1.0 mm). Furthermore, compound **2** was the most active metabolite, having a significant antimicrobial efficacy against *Rhizoctonia solani* (63.6 ± 1.1 mm), *Pseudomonas aeruginosa* (16.7 ± 0.6 mm), and *Alternaria alternate* (20.3 ± 0.6 mm) with MIC value at 15 µg/mL. In addition, compound **2** exhibited the most potency against hepatocellular (HepG-2) and skin (A-431) carcinoma cell lines with IC_50_ values of 107.16 µg/mL and 111.36 µg/mL, respectively.

**Conclusion:**

*Sphingomonas sanguinis* DM, a rhizosphere bacterium of *Datura metel*, was studied for its phytochemical and biological characteristics, resulting in the identification of two compounds with moderate antimicrobial and cytotoxic activities.

**Supplementary Information:**

The online version contains supplementary material available at 10.1186/s12906-024-04482-6.

## Background

Discovering unique and beneficial compounds is crucial since they enhance numerous aspects of human health [1]. As a result, bioactive natural products continue to influence contemporary medicine despite significantly increasing emphasis on synthetic products. Antibiotics are the cornerstone of modern medicine but are currently losing efficacy. Therefore, discovering novel natural products results from researching unexplored biodiversity niches [2]. With the growing need for food preservation and postharvest protection to control farm pests and pathogens, it is essential to find solutions that do not rely on synthetic agricultural chemicals and pesticides because they have safety and environmental issues [3]. This is where bioproducts, such as bacteria, come in, which have proven to be effective in extending storage life and protecting the environment while retaining the nutritional value of food [4]. These control agents are also significant in drug discovery due to their diverse structures and biological activities [5].

Bacterial and fungal infections are increasing tremendously, causing a huge global problem for the human race [6]. Antibiotic resistance has exacerbated the problem and the emergence of new pathogens that can spread widely [7]. One of contemporary science and technology’s most potent and influential contributions to managing infectious diseases is the discovery and development of antibiotics. Antibiotics are a diverse range of organic compounds that come from synthetic sources or can be produced by microbes. They function by inhibiting the growth or metabolic processes of other microorganisms [1]. These compounds possess a well-defined mode of action and impact crucial cellular processes such as DNA, RNA, protein, and cell wall formation. Additional research has been done to find powerful antimicrobial chemicals to combat pathogens, including *Staphylococcus* sp., *Mycobacterium tuberculosis*, and *Streptococcus* sp., that are resistant to drugs [1]. Scientific evidence suggests that natural compounds with cytotoxic activity found in nature can effectively eliminate tumor cells. Some of these compounds are currently utilized in chemotherapy treatments, while others have shown promising potential in preclinical research as agents that can combat tumors and metastasis [8]. Additionally, the discovery of new drugs has been aided by the random screening of natural products [9]. Specifically, studies indicate that *Sphingomonas* sp. can infiltrate and infect various mammalian cell lines. As a result of this infection, these cells exhibit a range of cytopathic effects, including vacuolation in the perinuclear zone, cytoplasmic granulation, and membrane blebbing [10].

*Datura metel* is a perennial herbaceous plant that is widely recognized for its manifold therapeutic properties and is frequently employed in traditional medicinal practices. Typically found in warmer regions, this plant is cultivated in tropical and subtropical areas for its stunning blossoms. With its numerous medicinal benefits, including antidiabetic, cytotoxic, antioxidant, anti-inflammatory, analgesic, antipyretic, neurological, wound-healing, and antispasmodic properties, it is no wonder that *Datura metel* is highly regarded in the world of traditional medicine [11]. Some of these properties are also believed to be linked to its potential antibacterial activity [12]. *Datura metel* has a rich history of traditional application in treating various ailments, including neurological and cardiovascular diseases, fever, catarrh, pain, diarrhea, skin diseases, chronic bronchitis, asthma, and digestive disorders. It is rich in several phytochemicals, including withanolides, daturaolone, datumetine, daturglycosides, ophiobolin A, baimantuoluoline A, and numerous others [13]. Additionally, *Datura metel* seeds are known to contain various substances that increase in concentration as the plant ages, such as alkaloids, tannins, cardiac glycosides, flavonoids, carbohydrates, amino acids, and phenolic substances [14].

The rhizosphere is home to a diverse microbial community that significantly impacts plant growth [15]. Rhizobacterial metabolites, including phenazines, phenolics, and pyrrole-type, are responsible for their antimicrobial activity [16]. *Sphingomonas*, a genus of rod-shaped bacteria, are known for their ability to survive in harsh environments and digest environmental toxins and industrial pollutants [17, 18]. *Sphingomonas* strains contain high concentrations of dihydrosphingosine, a long-chain sphingolipid, and polyamines. The genus has been extensively researched, with 139 known and published species [19, 20]. *Sphingomonas* has been obtained from various plant-based habitats, including the phyllosphere and rhizosphere [21, 22].

Based on the challenges above, much interest has been generated to explore the potential of investigating the biological effects of the secondary metabolites secreted from a rhizosphere strain, *Sphingomonas sanguinis* DM. In this study, the strain has been isolated from the rhizosphere of *Datura metel* and identified molecularly. In addition, two bioactive compounds were isolated, purified, and characterized according to various structure elucidation techniques. Lastly, various biological activities of these compounds, such as antimicrobial screening, MIC, and cytotoxicity, have been studied.

## Materials and methods

This research will look at the rhizosphere of *Datura metel* (family *Solanaceae*) and the purification and taxonomy of the isolated bacteria, followed by bacterium propagation, isolation, secondary metabolites purification, and lastly, analysis of isolated compounds, antimicrobial and cytotoxic activities.

### Materials

Gentamicin, ampicillin, and nystatin were obtained from the Micro Analytical Center, Faculty of Science, Cairo University. The analytical-grade *n*-hexane, ethyl acetate, dichloromethane, and methanol were procured from El Nasr Pharmaceutical Chemicals Co, Egypt. Top-quality dimethyl sulfoxide (DMSO), MTT, and trypan blue dye were obtained from Sigma (St. Louis, Mo., USA) for our experiment. Additionally, the fetal bovine serum, DMEM, RPMI-1640, HEPES buffer solution, L-glutamine, gentamicin, and 0.25% trypsin-EDTA were acquired from Lonza (Belgium).

### Plant material

*Datura metel* was collected from new Borg Elarab city, Alexandria, Egypt, in December 2021. The identification of the plant material was carried out by Dr. M. Gibali, Lecturer of plant taxonomy, NRC, Cairo, Egypt, and a specimen of the plant code (Pharm-EJUST 2021-05) was kept in the Pharmacognosy department, PharmD program, Egypt-Japan University of Science and Technology.

### Isolation of bacteria from the rhizosphere of *Datura metel*

Rhizosphere samples are collected by uprooting the root system and placing them in a cool box for transportation to the laboratory. They then ground one gram of soil using a sterile pestle and mortar. To isolate rhizosphere bacteria, serial dilutions (10X) were prepared, and 0.1 mL of each dilution ranging from 10^− 3^ to 10^− 5^ was distributed on tryptone soy agar plates [23]. The petri dishes were subjected to an overnight incubation period at 37 °C. Distinct colonies developed on the growing medium were selected for further purification. These colonies were then restreaked on fresh plates with tryptone soy agar medium to ensure purity and obtain single-cell colonies. Lastly, the bacterial cultures were grown at 37 °C for 24 h and preserved in 20% glycerol at -80 °C [24].

### DNA extraction, sequencing, and phylogenetic analysis of the selected bacterial strain

The bacterial isolate’s 16S rRNA gene underwent sequencing with the help of SolGent Company, located in Daejeon, South Korea. The bacteria were first cultivated on a nutrient agar medium for 24 h at 37 °C. A minute quantity of each isolate was carefully transferred into two mL sterile Eppendorf tubes and subsequently suspended in 100 µl of sterilized distilled water. The samples were then boiled at 100 °C for 15 min to extract the bacterial DNA. SolGent Company purified the extracted DNA using a SolGent purification bead. The polymerase chain reaction (PCR) technique was used to amplify the ribosomal rRNA gene (rDNA), including two universal bacterial primers (27F and 1492R) in the reaction mix. Electrophoresis was used to confirm the size of the purified PCR products (amplicons) using a nucleotide marker (100 base pairs) on a 1% agarose gel. The resulting bands were then eluted, and dideoxynucleotides (ddNTPs) were integrated into the reaction mixture for sequencing the bands in both the sense and antisense directions, using 27F and 1492R primers [25].

### Phylogenetic analysis

The sequences underwent analysis via the Basic Local Alignment Search Tool (BLAST) on the National Center of Biotechnology Information (NCBI) website. To create a phylogenetic tree or a dendrogram, the sequences were aligned using the ClustalX program (version 1.81) and the neighbor-joining (NJ) method [26].

### Large-scale fermentation and working up for extraction of secondary metabolites

A potent strain was initially carefully selected and utilized to produce a bacterial suspension. The suspension was then introduced into a 100 mL tryptone soy broth medium and cultivated for two days at a temperature of 37^o^C, producing a seed culture. For propagation, 5 mL of the seed culture was transferred aseptically into ten 1 L Erlenmeyer flasks, each containing 500 mL of broth medium. The flasks were left to incubate for 14 days under continuous shaking at 37^o^C [27, 28]. Following incubation, the bacterial cultures were sonicated for 30 min to break the cells. The resulting broth, which had a total volume of 5 L, was extracted by maceration in methanol (5 L). The methanol extract was subsequently filtered and concentrated *in vacuo*. The remaining residue was then suspended in water and extracted by ethyl acetate until exhaustion. Finally, the ethyl acetate extract was evaporated until completely dry and reserved for further purification.

### Isolation, purification, and characterization of the secondary metabolites

The obtained ethyl acetate portion (2.5 g) was fractionated using column chromatography (60 × 3 cm) packed with normal phase silica gel (Merck (Germany); 70–230 mesh). The column was eluted with a gradient mobile phase [DCM: MeOH (95:5)], and the fractions separated from the mixture were examined using TLC. The common fractions were collected, and the solvent was subjected to volatility using a rotatory evaporator under a vacuum and temperature not exceeding 40°C. The final purification of the isolated compounds was achieved using a preparative thin-layer chromatography technique (PTLC, DC-Alufolien, silica gel 60 matrix, Merck, Germany). The plates were eluted using a solvent system composed of *n*-hexane and ethyl acetate (9:1). The isolated zones were detected and marked under a UV lamp at wavelength 254 nm. The marked zones were scratched from the plates and extracted separately using a mixture of DCM and MeOH to yield compounds **1** and **2**. The purity of the isolated compound was tested on TLC using the same mobile phase, sprayed with anisaldehyde sulphuric acid reagent, and heated till complete visualization of the spots.

The Bruker Avance DRX instrument (Bruker, Rheinstetten, Germany) was utilized to record the 1D and 2D-NMR spectra, operating at 400 MHz (for ^1^H NMR) and 100.40 MHz (for ^13^C NMR). The solvents used for compounds **1** and **2** were deuterated chloroform (CDCl_3_) and methanol (CD_3_OD), respectively. Meanwhile, the XEVO TQD triple quadruple instrument (Waters Corporation, Milford, MA01757 U.S.A, mass spectrometer) was used to carry out the ESI-MS positive ion acquisition mode. For the ACQUITY UPLC-BEH C18 1.7 μm-2.1 × 50 mm Column, a solvent system of (A) water containing 0.1% formic acid and (B) acetonitrile was utilized with a flow rate of 0.2 mL/min. A spectrophotometer (UV-1800) Shimadzu was also used for characterization.

### Screening of the antimicrobial activities of the rhizosphere strain

#### Test microorganisms

Gram-positive bacteria (*Staphylococcus aureus* ATCC:13,565 and *Streptococcus mutans* ATCC:25,175) and Gram-negative bacteria (*Escherichia coli* ATCC:10,536, *Pseudomonas aeruginosa* ATCC:27,853 and *Klebsiella pneumonia* ATCC:10,031) were obtained from Micro Analytical Center, Faculty of Science, Cairo University. Fungal Phytopathogens (*Alternaria alternate* SCUF0000310, *Rhizoctonia solani* SCUF0000317, and *Fusarium oxysporum* SCUF000091) were obtained from the Agricultural Genetic Engineering Research Institute, Cairo University, Cairo.

### Antimicrobial activity

A comprehensive study was conducted to analyze the effectiveness of bacterial extract and its pure secondary metabolites against various human and phytopathogenic microorganisms. To observe the zones of inhibition in comparison with standard antibiotics, the study utilized the agar well diffusion method. The antibacterial activity of the compounds was tested in vitro using a Mueller-Hinton agar medium, while the antifungal activity was tested using a Sabouraud dextrose agar medium. Standard drugs such as Ampicillin and Gentamicin were used for Gram-positive and Gram-negative bacteria, respectively, whereas Nystatin was used for fungal strains. DMSO was used as a control solvent. The experiment involved puncturing the solidified media with wells of 6 mm diameter using a sterilized cork borer. Subsequently, 100 µL of the solution, comprising the compound under scrutiny, was added to each well of the plates. The plates were then incubated at 37 °C for a duration of 24 h to observe the antimicrobial properties. The experiment was repeated thrice to ascertain the precision, and the zones of inhibition were quantified in mm scale [29].

### Determination of MICs

A fresh colony of each bacterial strain was transferred into a tube containing 3–4 mL of a sterile broth medium for the antimicrobial susceptibility test. The tube was then incubated at 37 °C for 2–6 h, after which the resulting bacterial suspension was thoroughly mixed. It is important to note that the turbidity of the suspension should be at least as high as the turbidity of a McFarland Standard 0.5. The next step in the process involved dissolving the antimicrobial agent to be tested in 1 mL of DMSO, followed by a two-fold serial dilution (ranging from 4.37 to 280 µg/mL for compound **1** and 3.75 to 480 µg/mL for compound **2**) using a broth medium. A predetermined bacterial inoculum volume was added to each tube, and the tubes were incubated at 37 °C for 16–20 h (or 24–48 h in the case of a fungal inoculum). Finally, the antimicrobial agent’s minimum inhibitory concentration (MIC) was determined as the lowest concentration that prevented the visible growth of the tested isolate, as observed without any external assistance [30].

### Cytotoxicity

#### Mammalian cell lines

The cytotoxic assaying of compound **2** was carried out against two human breast cancer cell lines (MCF-7 and MDA-MB-231), human skin carcinoma (A-431), human hepatocellular cancer cell line (HepG-2), and human colorectal cancer cell line (HCT-116) that acquired from the American Type Culture Collection (ATCC, Rockville, MD).

### Cell culture

The cells were meticulously cultivated in RPMI-1640 medium, enriched with inactivated fetal calf serum (10%) and gentamycin (50 µg/mL). To maintain ideal growth conditions, they were housed in a humidified atmosphere at 37ºC and 5% CO_2_ and subcultured 2–3 times per week to ensure uniform development.

### Cytotoxicity assessment using viability assay

For antitumor assays, tumor cell lines were added to Corning® 96-well tissue culture plates at a concentration of 5 × 10^4^ cells per well. The cells were incubated for 24 h before exposure to tested compounds in the 96-well plates with three replicates. The compounds were used at varying concentrations (ranging from 0 to 500 µg/mL), as detailed in additional file 1. To ensure quality control, six vehicle controls containing media or 0.5% DMSO were included for each 96-well plate. After a 24-hour incubation period, cell viability was assessed using the MTT test. The media present in the 96-well plates was accurately extracted and substituted with 100 µl of fresh RPMI 1640 culture medium, devoid of phenol red. Next, 10 µl of 12 mM MTT stock solution, including the untreated controls, was added to each well. This solution contained 5 mg of MTT in 1 mL of PBS. The 96-well plates were incubated at 37 °C with a 5% concentration of CO_2_ for four hours. Following incubation, 85 µL of media was extracted from the wells and substituted with 50 µL of DMSO. The wells were then completely blended using a pipette and further incubated at the same temperature for 10 min to determine the number of viable cells. The study used a microplate reader (SunRise, TECAN, Inc, USA) to measure the optical density at 590 nm and determine viable cell count. To determine the viability percentage of tumor cells, the formula [(ODt/ODc)] x100% was used, where ODt represents the average optical density of wells treated with the tested sample, and ODc represents the average optical density of untreated cells. Survival curves were generated for each tumor cell line based on the provided data following exposure to a specific compound. The GraphPad Prism software (San Diego, CA, USA) was employed to estimate the concentration (IC_50_) at which toxic effects are observed in 50% of intact cells by analyzing the dose-response curve for each concentration [31].

### Statistical analysis

An analysis of the antimicrobial study data was conducted utilizing a one-way analysis of variance (ANOVA). Subsequently, Duncan’s multiple comparisons test was employed in conjunction with the SPSS package version “22” for Windows. For the in vitro cytotoxic assay, indicated compound concentrations were tested twice in triplicate. The statistical analysis utilized one-way ANOVA, along with Tukey’s post hoc test, to make a comparison between mean cell proliferation at indicated concentrations and DMSO-treated control cells.

## Results

### Isolation and taxonomical characterization of bioactive compounds producing strain

This investigation examined the growth of the rhizosphere strain obtained from the rhizosphere of *Datura metel* on tryptone soy agar plates after 12–24 h. It was isolated and cultivated, and pure cultures were kept at -80 °C in glycerol (20%). According to morphological criteria, the cells were Gram-negative, yellow-pigmented, strictly aerobic, motile, non-spore-forming, and rod-shaped. The potent bacterial strain was identified through 16S rRNA gene analysis. The bacterial DNA was extracted, and the 16S rDNA sequence was amplified and sequenced using universal primers. The resulting 16S rDNA sequence was compared to the non-redundant BLAST database to obtain the sequences with the highest similarity. The BLAST algorithm found that the selected bacterium’s 16S rDNA sequence had a very high similarity percentage (99.61%) with the sequence of *Sphingomonas sanguinis* NBRC 13937 with a reasonably high score and e-value of zero (Additional file 2). Two strains with similar morphological characteristics are Gram-negative, rod-shaped, chemoheterotrophic, strictly aerobic bacteria and produce yellow-pigmented colonies. They can also survive in harsh conditions. The genomic structure of *Sphingomonas* is characterized by a distinctive composition that includes major 2-OH fatty acids, homospermidine as the primary polyamine, and signature nucleotide bases within the 16S rRNA gene.

**Fig. 1:**
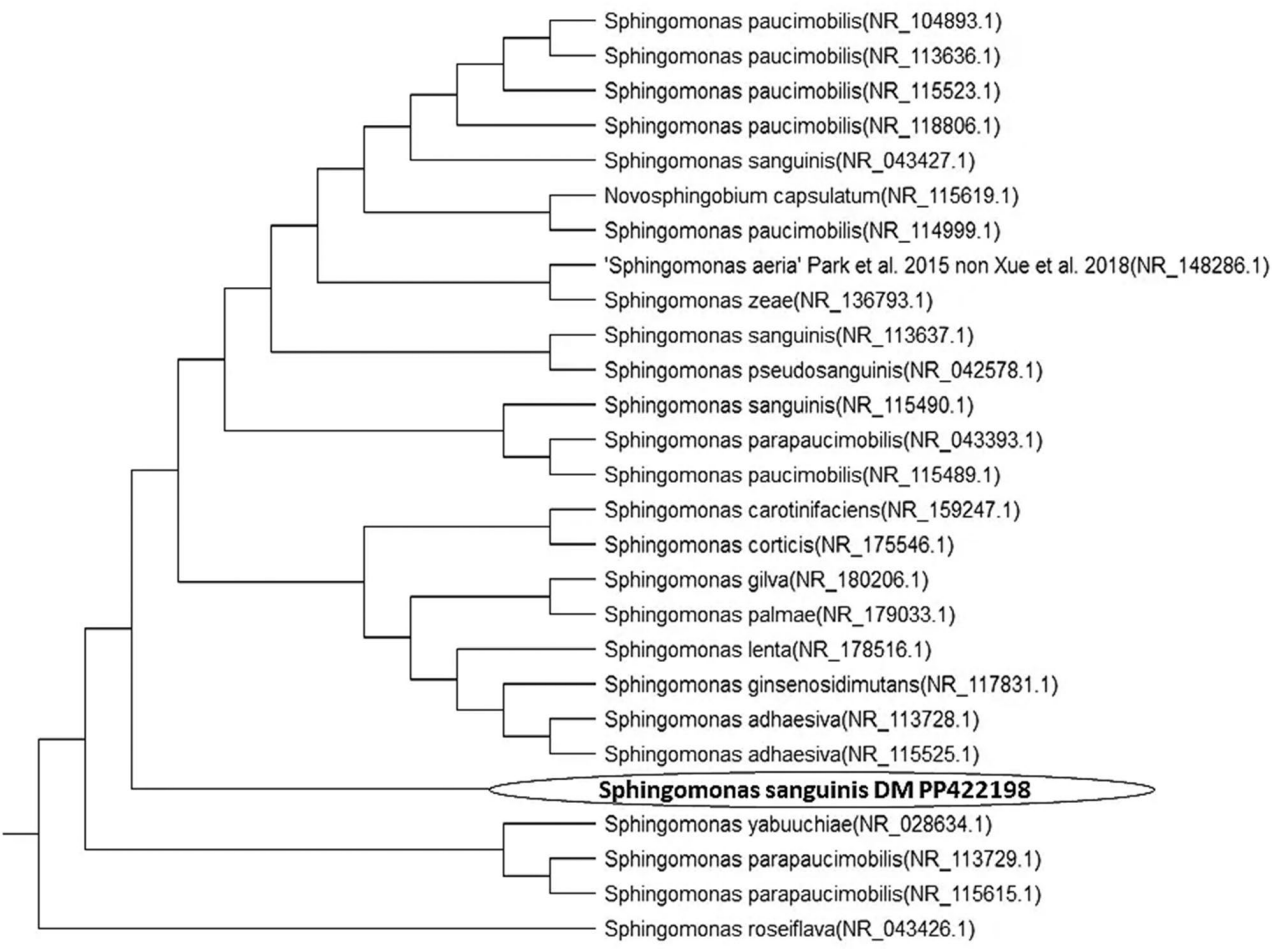
The Phylogenetic tree (1000 bootstrap replicates) depicts the relation between the 16S rDNA sequence of the rhizosphere *Sphingomonas sanguinis* DM and its possible homologous sequences accessed from the GeneBank

The phylogenetic relationship was established by employing a freely available alignment program called ClustalX. This program used the neighbor-joining algorithm to connect the homologous sequences obtained by the BLAST program. The sequence data for strains that showed many similarities with our strain was obtained from the gene bank. This connection is necessary to determine the species linked to the selected bacterium. The evolutionary relationship is shown as a dendrogram (Fig. 1), demonstrating a distinct rooted evolution.

### Structure elucidation of the isolated compounds

#### Compound 1

This compound (37.8 mg) has R_f_ = 0.68 using a solvent system composed of *n*-hexane: ethyl acetate (9:1) and visualized under short wavelength UV light, UV absorbance λ_max_ (chloroform) 234.50 nm. This compound exhibits m/z = 279 [M^+^+1] and 301 [M^+^+Na] ESI positive ion mode corresponding to a molecular formula C_16_H_22_O_4_. ^1^H NMR spectra of this compound showed two multiple peaks at δ 7.73 and δ 7.54, respectively, corresponding to ortho disubstituted benzene ring and spin coupling system AA’BB’ in addition to two hydroxy methylene groups at δ 4.22 (m), four aliphatic methylene protons at δ 1.39 (m) and two methyl groups at δ 0.94 (m). HH COSY spectra showed one critical cross peak between H-1’, H-2’, and H-1’’, H-2’’, respectively.

**Fig. 2:**
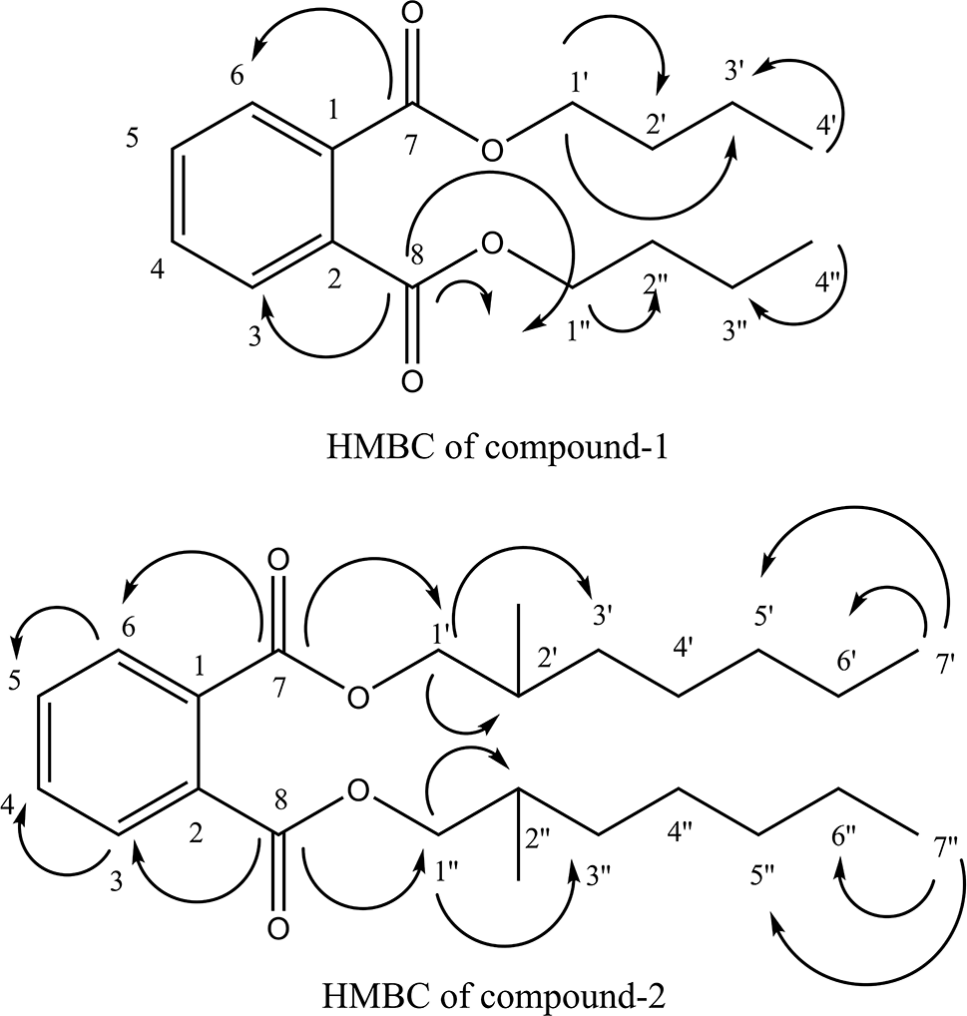
Structure of compounds **1** and **2**

The ^13^C-NMR APT spectra revealed the presence of 2 quaternary carbons at δ 167.71 of carboxyl function and δ128.84, respectively, corresponding to C-7, C-8, and C-1, C-2. The two oxy-methylene groups appear at δ 65.55 and 4 aliphatic methylene carbons, each pair at δ 30.58 and δ 19.18, respectively. The two methyl groups appear at δ 13.72. The HMBC spectra of compound **1** showed many useful cross-peaks between (C-7, H-6), (C-8, H3), (C-7, H1’), (C-8, H1”), (C-4’, H-3’), (C-4”, H-3”), (C-1’, H-2”) (Fig. 2). From the previous data, compound **1** could be identified as n-dibutyl phthalic acid [32] (Additional file 3: Figs. S1–S9).

### Compound 2

This compound (62.1 mg) has R_f_ = 0.81 using a solvent system composed of *n*-hexane: ethyl acetate (9:1) and visualized under short wavelength UV light, UV absorbance λ_max_ (chloroform) 250.00 nm. This compound exhibits m/z = 413 [M^+^+Na] ESI positive ion mode corresponding to a molecular formula C_24_H_38_ O_4_. ^1^H NMR spectra of this compound showed two multiple peaks at δ 7.73 and δ 7.61 respectively corresponding to ortho disubstituted benzene ring and spin coupling system AA’BB’ in addition to two hydroxy methylene groups at δ 4.22 (m), four aliphatic methylene protons at δ 1.39 (m) and two methyl groups at δ 0.94 (m) in addition to one methine proton at δ 1.69 (s). HH COSY spectra showed one critical cross peak between the oxy-methylene protons of H-1’ H-2’ and the methine proton of H-1’’ and H-2’’, respectively.

The ^13^C NMR APT spectra revealed the presence of 2 quaternary carbons at δ 167.89 of carboxyl function and δ 132.22, respectively, corresponding to C-7, C-8, and C-1, C-2. The two oxy-methylene groups appear at δ 67.74 in addition to 8 aliphatic methylene carbons, each pair at δ 30.24, δ 28.76, δ 23.58, and δ 22.66. The two methyl groups appear at δ 13.10 and δ 10.11, respectively. The location of a methyl group at C2’, C2’’was deduced from the cross peak between the methine proton at C-2’, C2” and the oxy-methylene carbon at C-1’ and C-1”. The HMBC spectra of compound **2** showed many useful cross-peaks between (C-7, H-6), (C-8, H3), (C-7, H1’), (C-8, H1”), (C-4’, H-3’), (C-4”, H-3”), (C-1’, H-2”), (C-7’, H-6’)., (C-7”, H-6”)., (C-7’, H-5’)., (C-7”, H-5”) (Fig. 2). From the previous data, compound **2** could be identified as Bis (2-methyl heptyl) phthalate [33] (Additional file 4: Figs. S10–S18). It is worth noting that the isolation of the two compounds is the first report from the genus *Sphingomonas*.

### Biological activities

#### Antimicrobial screening

The crude extract isolated from *Sphingomonas sanguinis* and its pure secondary metabolites were screened and showed considerable antimicrobial activity. Following the allotted period for incubation, clear zones against the tested bacteria and fungi were observed and measured in millimeters. The mean inhibition zones produced by the various substances fluctuated between 10.6 and 63.6 mm (Table 1). Based on the agar well diffusion method, the extract of the bacterial strain could exhibit antibacterial action, inhibiting at least two of the bacterial pathogens. It showed its activity against gram-positive bacteria, moderate activity against *Streptococcus mutans* (12.6 ± 0.6 mm), and weak activity against *Staphylococcus aureus* (10.6 ± 0.6 mm), in contrast to several standard antibiotics. However, there was no evidence of any possible activity against gram-negative bacteria or fungal pathogens by the bacterial extract.

**Table 1:** Antimicrobial activities of bacterial extract and its pure metabolites recovered from *Sphingomonas sanguinis* DM

Microorganism	Sample			
	Zone of inhibition (mm)			
	Crude extract	Compound 1	Compound 2	Standard antibiotic
Gram negative bacteria				Gentamicin
*Escherichia coli* (ATCC:10,536)	NA	NA	10.7 ± 0.6	27 ± 1.0
*Klebsiella pneumonia* (ATCC:10,031)	NA	11.0 ± 1.0	11.6 ± 0.7	25.3 ± 0.7
*Pseudomonas aeruginosa* (ATCC:27,853)	NA	NA	16.7 ± 0.6	28 ± 1.0
Gram-positive bacteria				Ampicillin
*Staphylococcus aureus* (ATCC:13,565)	10.6 ± 0.6	NA	11.3 ± 0.6	21.3 ± 0.7
*Streptococcus mutans* (ATCC:25,175)	12.6 ± 0.6	NA	11.7 ± 0.6	28.3 ± 0.7
Fungi				Nystatin
*Alternaria alternate* (SCUF0000310)	NA	56.3 ± 10.6	20.3 ± 0.6	21 ± 0.5
*Rhizoctonia solani* (SCUF0000317)	NA	NA	63.6 ± 1.1	19 ± 0.5
*Fusarium oxysporum* (SCUF000091)	NA	21.3 ± 1.5	NA	15 ± 0.5

Values are means of three replicates ± standard deviation (SD)

**Fig. 3:**
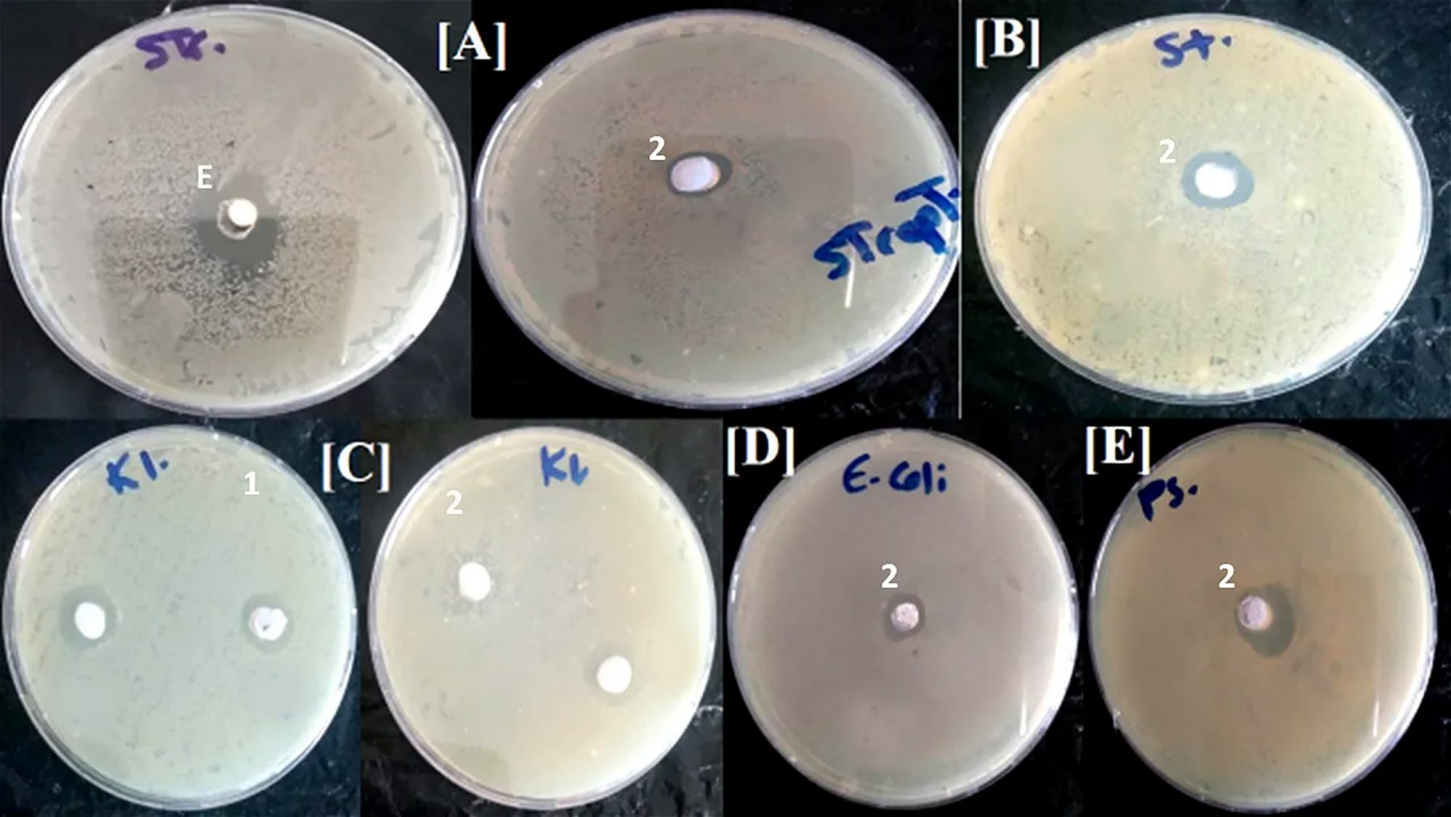
Antibacterial activities of bacterial extract (E) and its pure metabolites (**1** and **2**) isolated from the rhizosphere of *Datura metel* against [**A**] *Streptococcus mutans*, [**B**] *Staphylococcus aureus*, [**C**] *Klebsiella pneumonia*, [**D**] *Escherichia coli*, [**E**] *Pseudomonas aeruginosa*

**Fig. 4:**
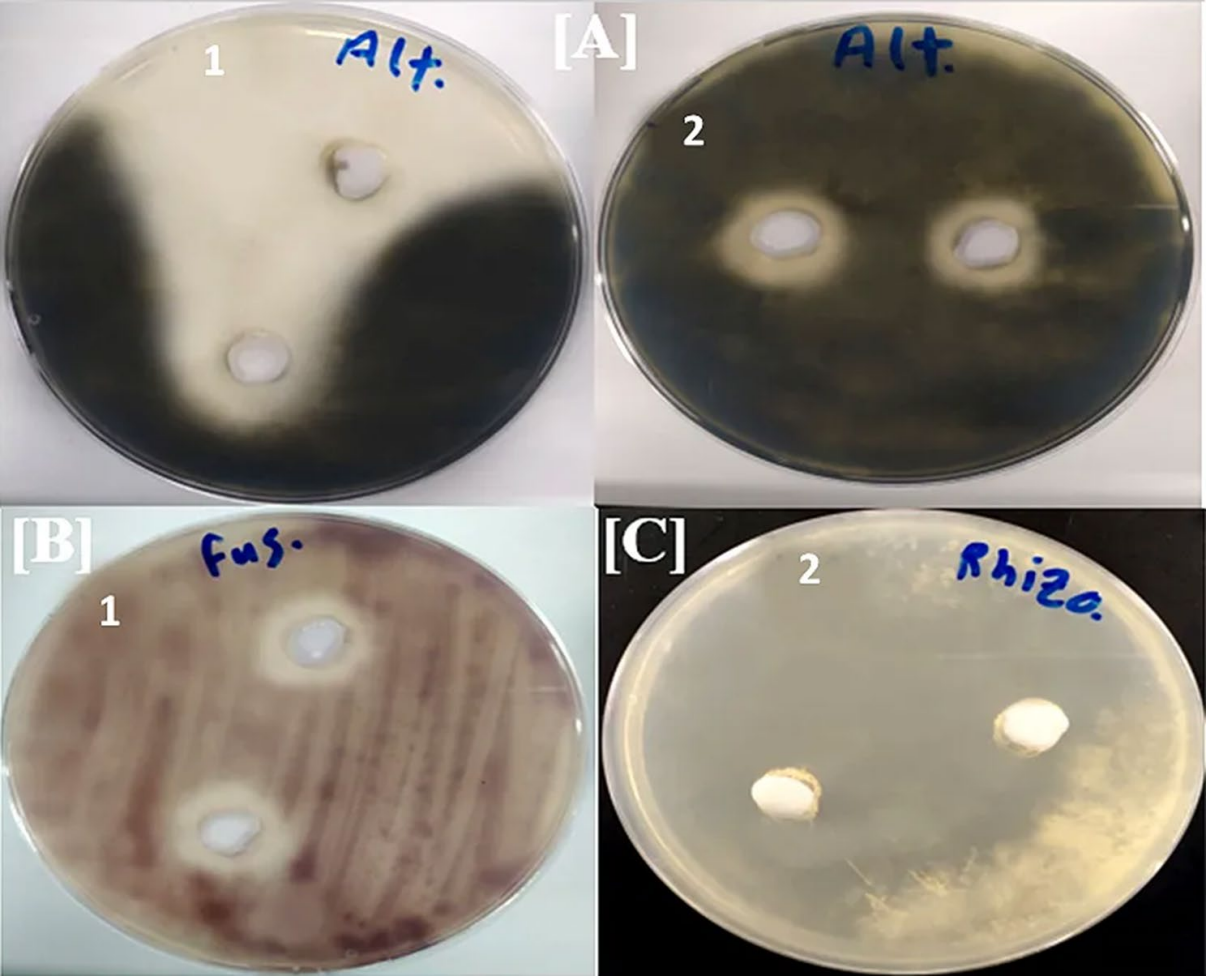
Antifungal activities of bacterial extract (E) and its pure metabolites (**1** and **2**) isolated from the rhizosphere of *Datura metel* against [**A**] *Alternaria alternate*, [**B**] *Fusarium oxysporum*, [**C**] *Rhizoctonia solani*

In contrast, pure secondary metabolites could exhibit antimicrobial activity against some pathogenic microbes, inhibiting at least three tested pathogens. Compound **2** was the most active metabolite, indicating significant antimicrobial activity against pathogenic fungi, such as *Rhizoctonia solani* (63.6 ± 1.1 mm) and *Alternaria alternate* (20.3 ± 0.6 mm) and pathogenic bacteria such as *Pseudomonas aeruginosa* (16.7 ± 0.6 mm). In addition, compound **1** was weakly active against *Klebsiella pneumonia* (11.0 ± 1.0 mm) but displayed a striking inhibitory effect against phytopathogenic fungi, such as *Alternaria alternate* (56.3 ± 10.6 mm) and *Fusarium oxysporum* (21.3 ± 1.5 mm) (Figs. 3 and 4). To our knowledge, compound **2** has been examined for the first time for its ability to target the tested pathogens.

### Minimum inhibitory concentrations (MICs)

MICs of pure secondary metabolites ranged from 15 to 240 µg/mL against the tested pathogens. The present study showed high activity of different metabolites against pathogenic microorganisms. Table 2 showed that the most active compound with the lowest MICs was compound **2**, having a MIC value of 15 µg/mL against *Alternaria alternate*. In contrast, compound **1** was the least active and had a MIC value of 17.5 µg/mL against *Fusarium oxysporum*. It is worth mentioning that this study is the first to test the MIC value of compound **2** against different pathogens.

**Table 2:** MICs of pure secondary metabolites recovered from *Sphingomonas sanguinis* DM against the tested organisms

Microorganism	Sample		
	MICs (µg/mL)		
	Compound 1	Compound 2	Standard antibiotic
Gram-negative bacteria			Gentamicin
*Escherichia coli* (ATCC:10,536)	-	240	31.25
*Klebsiella pneumonia* (ATCC:10,031)	35	240	62.5
*Pseudomonas aeruginosa* (ATCC:27,853)	-	30	125
Gram-positive bacteria			Ampicillin
*Staphylococcus aureus* (ATCC:13,565)	-	120	62.5
*Streptococcus mutans* (ATCC:25,175)	-	240	62.5
Fungi			Nystatin
*Alternaria alternate* (SCUF0000310)	35	15	25
*Rhizoctonia solani* (SCUF0000317)	-	30	22.3
*Fusarium oxysporum* (SCUF000091)	17.5	-	32.4

### Cytotoxicity screening

**Fig. 5:**
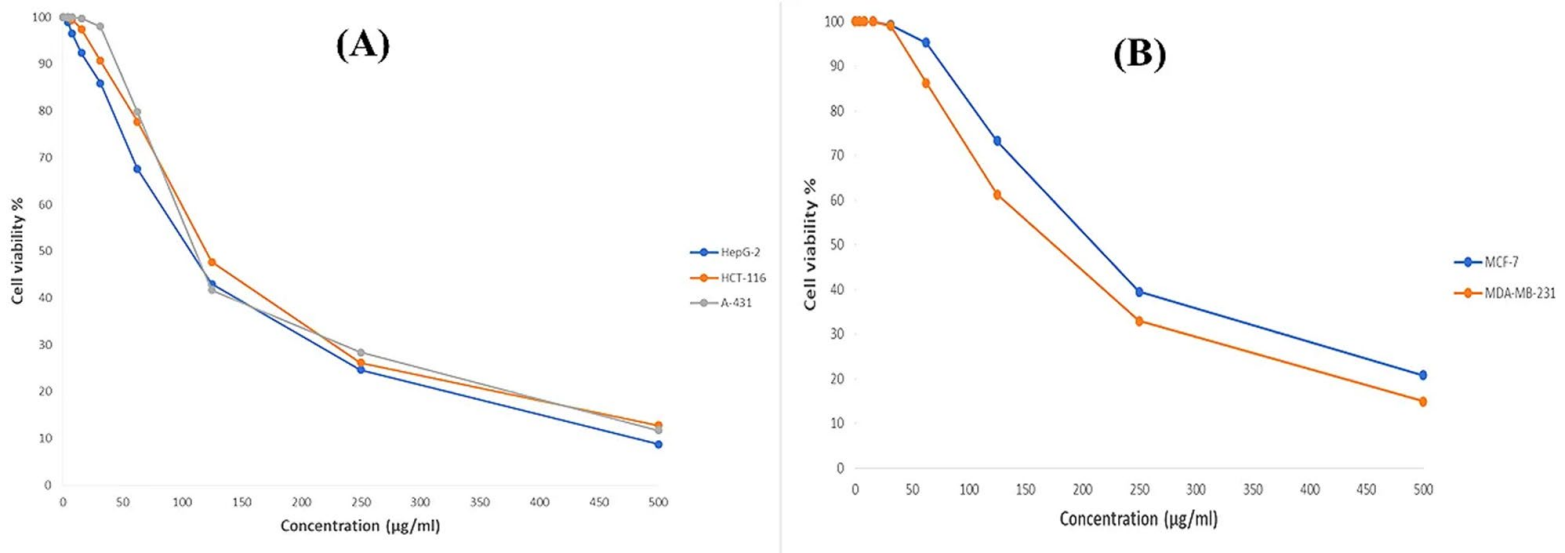
(**A**) Cytotoxic activity shown as a % Cell Viability of hepatocellular (HepG-2), skin (A-431), and colorectal (HCT-116) at different concentrations (0–500 µg/mL) of compound **2**. (**B**) Dose-response of compound (2) against two breast cancer cell lines (MDA-MB-231, MCF-7) after treatment for 48 h

The in vitro anticancer activities of compound **2**, Bis-(2-methyheptyl)-phthalate, were evaluated against hepatocellular (HepG-2), colorectal (HCT-116), skin (A-431) and two breast cancer cell lines (MDA-MB-231, MCF-7). Cells were treated with compound **2** for 48 h at different concentrations (0–500 µg/mL), while DMSO was used as blank. The results (Fig. 5) were expressed in terms of reducing yellow MTT to purple formazan by metabolically viable cells, and the IC_50_ (µg/mL) is indicated in Table 3.

**Table 3:** IC_50_ (µg/mL) of antitumor activity of compound **2** against hepatocellular (HepG-2), colorectal (HCT-116), skin (A-431) and two breast cancer cell lines (MDA-MB-231, MCF-7)

Cell line	IC_50_ (µg/mL)
HepG-2	107.16±3.47
A-431	111.36±0.73
HCT-116	120.14±4.64
MDA-MB-231	174.45±6.05
MCF-7	211.12±9.74

Values are means of three replicates ± standard deviation (SD)

The results showed that compound **2** exhibited the most potency against hepatocellular (HepG-2) and skin (A-431) carcinoma cell lines with IC_50_ values of 107.16 µg/mL and 111.36 µg/mL, respectively, at which 50% of the HepG-2 and A-431 cells are viable, announcing a gradual cytotoxic potency with about 8.75% and 11.71% cell viability at concentration of 500 µg/mL, respectively. In addition, it was found that compound **2** was moderately cytotoxic towards the colorectal cell line (HCT-116) with an IC_50_ value of 120.14 µg/mL (with about 26.21% viability) at a concentration of 250 µg/mL. Meanwhile, the lowest cytotoxicity was examined when compound **2** was separately tested against two breast cancer cell lines (MDA-MB-231, MCF-7) that showed IC_50_ values of 174.45 µg/mL and 211.12 µg/mL and viability of 32.92% and 39.51% at the same concentration, respectively (Fig. 5).

## Discussion

It has been shown that all of the sequences originate from a single ancestor that later split into two distinct clusters, each comprised of a unique strain of *Sphingomonas* sp. In light of the findings, the bacterium under investigation has been identified and designated as *Sphingomonas sanguinis* DM. The accession number PP422198, related to the 16S rDNA sequence of a particular bacterium, has been successfully registered in the GenBank database.

A wide variety of plant-beneficial bacteria has been shown to colonize the roots and rhizosphere of *Datura metel* and belong to a wide variety of genera. Among them, *Sphingomonas sanguinis* DM isolated in the current study can survive in harsh conditions. The soil microbiota might be necessary for nutrient transformations and other critical plant activities. To research the variety of the microbiota, some microorganisms associated with the rhizosphere of *Datura stramonium* L. have been conserved [34]. The genus *Sphingomonas* is considered a rhizobacterium as it was earlier isolated from the rhizosphere of different plants. The taxonomical characterization in the current study agrees with studies that recently exhibited various species of *Sphingomonas.* For instance, rhizosphere soil samples were obtained from rice fields (*Oryza sativa* L.), and *Sphingomonas oryziterrae* sp. nov. and *Sphingomonas jinjuensis* sp. nov. were isolated as new species of the genus [21]. Also, a polyphasic taxonomic approach was utilized to characterize *Sphingomonas quercus* sp. nov., which was isolated from the rhizosphere soil of *Quercus mongolica* [19].

According to antimicrobial screening, the crude total extract and compound **2** of *Sphingomonas sanguinis* DM demonstrated efficacy against Gram-positive bacteria. In contrast, the whole extract was not as effective against Gram-negative bacteria and phytopathogens. Pure compounds **1** and **2** exhibited the highest activity level against phytopathogenic microorganisms, including *Alternaria alternate* and *Rhizoctonia solani*, considered the most susceptible pathogens. However, it was proved that *Escherichia coli, Pseudomonas aeruginosa*, and *Fusarium oxysporum* were the most resistant pathogenic strains. The obtained bacterial extract may combat other microbes not included in the testing. The variance in sensitivity between Gram-positive and Gram-negative bacteria is due to structural differences. Gram-negative bacteria possess an outer membrane containing lipopolysaccharide components, making the cell wall impermeable to lipophilic solutes. In contrast, Gram-positive bacteria are more vulnerable because they only have an outer peptidoglycan layer, which is not an effective permeability barrier [35].

*Sphingomonas sanguinis* DM is a promising bioactive strain, showing a wide range of inhibition zones against various human and phytopathogenic microorganisms. The strain can contain antagonistic properties, which are essential for suppressing the growth of pathogens. The inhibitory effect can be shown on the cell wall or DNA of the pathogen. The bioactive strain could also block enzymes responsible for metabolic activity in the pathogen. Thus, *Sphingomonas sanguinis* DM may effectively control pathogens to protect the plant against phytopathogens. Research findings have indicated the role of *Sphingomonas* as a biocontrol agent that demonstrated different antimicrobial activities. For example, *Sphingomonas* has been tested for its potential to create a variety of antimicrobial chemicals that are connected with the rhizosphere and the phyllosphere [36]. *Sphingomonas* spp. can defend plants against *Xanthomonas campestris* pv. *campestris* (Xcc) that cause black rot for crucifers [37], so they may also be effective against various foliar pathogens. Ji and Wilson [38] showed that it is more likely for a bacterial strain to be effective against a plant pathogen if it has a higher degree of nutritional similarity with the pathogen. Di-n-butyl phthalate, diethyl phthalate, dimethyl phthalate, di-(2-ethylhexyl) phthalate, di-isobutyl phthalate, etc., are the most common dominant phthalic acid esters (PAEs) found in natural sources. According to a recent study, di-(2-ethyl hexyl) phthalate has been found to exhibit significant antibacterial properties against various strains of methicillin-resistant *Staphylococcus aureus*, with a range of zone of inhibition between 21.5 and 30.7 mm [39].

In the current investigation, the MIC values (Table 2) were discovered to be lower than those of the data obtained from previous studies. For instance, various metabolites, including sattabacin-like and xenocyloin B, which were isolated from *Sphingomonas*, demonstrated MIC values below 32 µg/mL against a panel of clinical *Acinetobacter baumannii* strains [40]. In addition, di-butyl phthalate, which was derived from *Streptomyces albidoflavus*, has been shown to suppress the growth of Gram-positive bacteria (*B. subtilis*, MIC 84 µg/ml), Gram-negative bacteria (*E. coli*, MIC 53 µg/ml; *S. typhi*, MIC 76 µg/ml), yeast (*S. cerevisiae*, MIC 92 µg/ml), and filamentous fungi (*A. niger*, MIC 98 µg/ml and *C. pallescens*, MIC 117 µg/ml) [41]. The antibacterial activity of di-(2-ethylhexyl) phthalate isolated from *Calotropis gigantean* against *B. subtilis* has a MIC value of 32 µg/mL [42]. The development of drugs with economic feasibility requires MIC values that are relatively low to be successful.

In the current study, bis (2-methyl heptyl) phthalate (compound **2**), which was isolated from the crude extract of a rhizosphere strain, *Sphingomonas sanguinis* DM, was screened for the first time to evaluate its cytotoxic activities as it can act as a growth inhibitor against the tested human cancer cell lines. The IC_50_ and MIC exhibit a strong correlation according to the present results in both assays, indicating that the mechanisms behind antibacterial efficacy and cytotoxicity are similar. The increased drug concentration is responsible for improved antimicrobial efficacy against various pathogens and significant cytotoxicity towards cancer cells. Numerous studies indicate that certain phthalate esters (PAEs) in microbial resources possess cytotoxic properties that could combat multiple cancer cell lines [43]. The toxicity profiles of PAEs differ based on their chemical structures. Phthalic acid esters (PAEs) have been found to have detrimental effects on human and animal health. These effects include disruption of the endocrine system, inhibition of enzymes in the testes, malformation of the reproductive tract, weight loss, and negative effects on brain development [44]. Di (2-ethyl hexyl) phthalate (DEHP) is one type of PAEs, and at high doses, it can inhibit the growth of tumor cells [45].

The most related derivative of phthalate ester, bis (6-methylheptyl) phthalate, was isolated from *Phyllanthus pulcher* and displayed anticancer activity against MCF-7 cell lines with an IC_50_ value of 26.98 µg/ml [46], which is remarkably lower than that of compound **2** isolated from *Sphingomonas sanguinis* DM extract. As a result, compound **2** has the potential to be a unique medication against malignant human cancer cells. It is also found that anthropogenic derivatives of phthalate esters, particularly those with low molecular weight, are potentially cytotoxic to living systems [43]. For instance, bis-(2-ethyl heptyl) phthalate inhibited cathepsin B with an IC_50_ value of 121.22 µg/ml, causing neuroblastoma cell death [47] in comparison with compound **2** presented in this study that exhibited a slight decrease in IC_50_ values against HepG-2 and A-431 cell lines.

## Conclusion

Two bioactive metabolites (compound **1**, n-dibutyl phthalic acid) and (compound **2**, Bis (2-methyl heptyl) phthalate) were isolated from the rhizospheric *Sphingomonas sanguinis* DM. NMR data analysis illustrates the structure of compounds (**1** and **2**) obtained from the bacterial strain. Biologically, compound **2** is potentially active against *Rhizoctonia solani*, *Alternaria alternate*, and *Pseudomonas aeruginosa*. Also, it displayed outstanding cytotoxicity against hepatocellular (HepG-2) and skin (A-431) carcinoma cell lines. The most active compound with the lowest MICs was compound **2** against *Alternaria alternate*. 16S rRNA gene sequence has been recorded in the NCBI GenBank database under accession number PP422198 and identified as *Sphingomonas sanguinis* DM.

### Electronic supplementary material

Below is the link to the electronic supplementary material.

Supplementary Material 1

Supplementary Material 2

Supplementary Material 3

Supplementary Material 4

## Data Availability

All the data generated or analyzed in the study is included in this article and supplementary file itself.

## Abbreviations

DCM: Dichloromethane
MeOH: Methanol
PAEs: Phthalic acid esters
HepG-2: Hepatocellular cancer cell line
A-431: Skin cancer cell line
HCT-116: Colorectal cancer cell line
MDA-MB-231: Breast cancer cell line
MCF-7: Breast cancer cell line
Xcc: *Xanthomonas campestris* pv. *campestris*
