# Correction: Phytochemical and biological assessment of secondary metabolites isolated from a rhizosphere strain, *Sphingomonas sanguinis* DM of *Datura metel*

**DOI:** 10.1186/s12906-024-04543-w

**Published:** 2024-06-18

**Authors:** Mohamed A. Awad, Sherif F. Hammad, Samir F. El-Mashtoly, Bahig El-Deeb, Hesham S. M. Soliman

**Affiliations:** 1 Biotechnology Program, Institute of Basic and Applied Science, Egypt Japan University of Science and Technology (E-JUST), New Borg El-Arab City, Alexandria 21934 Egypt; 2 Botany and Microbiology Department, Faculty of Science, Sohag University, Sohag, 82524 Egypt; 3 Department of Pharmaceutical Chemistry, Faculty of Pharmacy, Helwan University, Helwan, Cairo 11795 Egypt; 4 Department of Pharmacognosy, Faculty of Pharmacy, Helwan University, Helwan, Cairo 11795 Egypt; 5 PharmD Program, Egypt-Japan University of Science and Technology (E-JUST), New Borg El- Arab City, Alexandria 21934 Egypt

**Correction: BMC Complement Med Ther 24, 205 (2024)**

10.1186/s12906-024-04482-6

Following publication of the original article [1], the authors reported an error in affiliations.

The incorrect affiliations are:

^1^ Biotechnology Program, Institute of Basic and Applied Science, Egypt Japan University of Science and Technology (E-JUST), New Borg El-Arab City, Alexandria 21,934, Egypt.

^2^ Botany and Microbiology Department, Faculty of Science, Sohag University, Sohag 82,524, Egypt.

^3^ PharmD Program, Egypt-Japan University of Science and Technology (E-JUST), New Borg El-Arab City, Alexandria 21,934, Egypt.

^4^ Department of Pharmaceutical Chemistry, Faculty of Pharmacy, Helwan University, Helwan, Cairo, Egypt.

^5^ Department of Pharmacognosy, Faculty of Pharmacy, Helwan University, Helwan, Cairo, Egypt.

The correct affiliations are:

^1^Biotechnology Program, Institute of Basic and Applied Science, Egypt Japan University of Science and Technology (E-JUST), New Borg El-Arab City, Alexandria 21,934, Egypt.

^2^Botany and Microbiology Department, Faculty of Science, Sohag University, Sohag 82,524, Egypt.

^3^Department of Pharmaceutical Chemistry, Faculty of Pharmacy, Helwan University, Helwan, Cairo 11,795, Egypt.

^4^Department of Pharmacognosy, Faculty of Pharmacy, Helwan University, Helwan, Cairo 11,795, Egypt.

^5^PharmD Program, Egypt-Japan University of Science and Technology (E-JUST), New Borg El-Arab City, Alexandria 21,934, Egypt.

The affiliations in the author group has been updated above and the original article [1] has been corrected.
