# Supplementary material for: Phytochemical and biological assessment of secondary metabolites isolated from a rhizosphere strain, Sphingomonas sanguinis DM of Datura metel
Source: BMC Complement Med Ther. 2024 May 25;24:205. doi: 10.1186/s12906-024-04482-6 (PMC11128111; doi:10.1186/s12906-024-04482-6)
Supplement: Supplementary file 1 — Supplementary Material 1 [file 12906_2024_4482_MOESM1_ESM.docx]

**Phytochemical and Biological Assessment of Secondary Metabolites Isolated from a Rhizosphere Strain, *Sphingomonas sanguinis* DM of *Datura metel***

Mohamed A. Awad^1,2^

mohamed.abo-elfadl@ejust.edu.eg

Sherif F.Hammad^3,5^

sherif.hammad@ejust.edu.eg

Samir F. El-Mashtoly^1^

samir.elmashtoly@ejust.edu.eg

Bahig El-Deeb^2^

bahig1978@gmail.com

Hesham S. M. Soliman^4,5*^

[hesham.soliman@ejust.edu.eg](mailto:hesham.soliman@ejust.edu.eg)

^1^Biotechnology Program, Institute of Basic and Applied Science, Egypt-Japan University of Science and Technology (E-JUST), New Borg El-Arab City, Alexandria 21934, Egypt

^2^Botany and Microbiology Department, Faculty of Science, Sohag University, Sohag 82524, Egypt

^3^Department of Pharmaceutical Chemistry, Faculty of Pharmacy, Helwan University, Helwan, Cairo 11795, Egypt

^4^Department of Pharmacognosy, Faculty of Pharmacy, Helwan University, Helwan, Cairo 11795, Egypt

^5^PharmD Program, Egypt-Japan University of Science and Technology (E-JUST), New Borg El-Arab City, Alexandria 21934, Egypt

* Corresponding author: [hesham.soliman@ejust.edu.eg](mailto:hesham.soliman@ejust.edu.eg)

**Cytotoxicity Results of Compound 2**

1. **Evaluation of cytotoxicity against A-431 cell line**


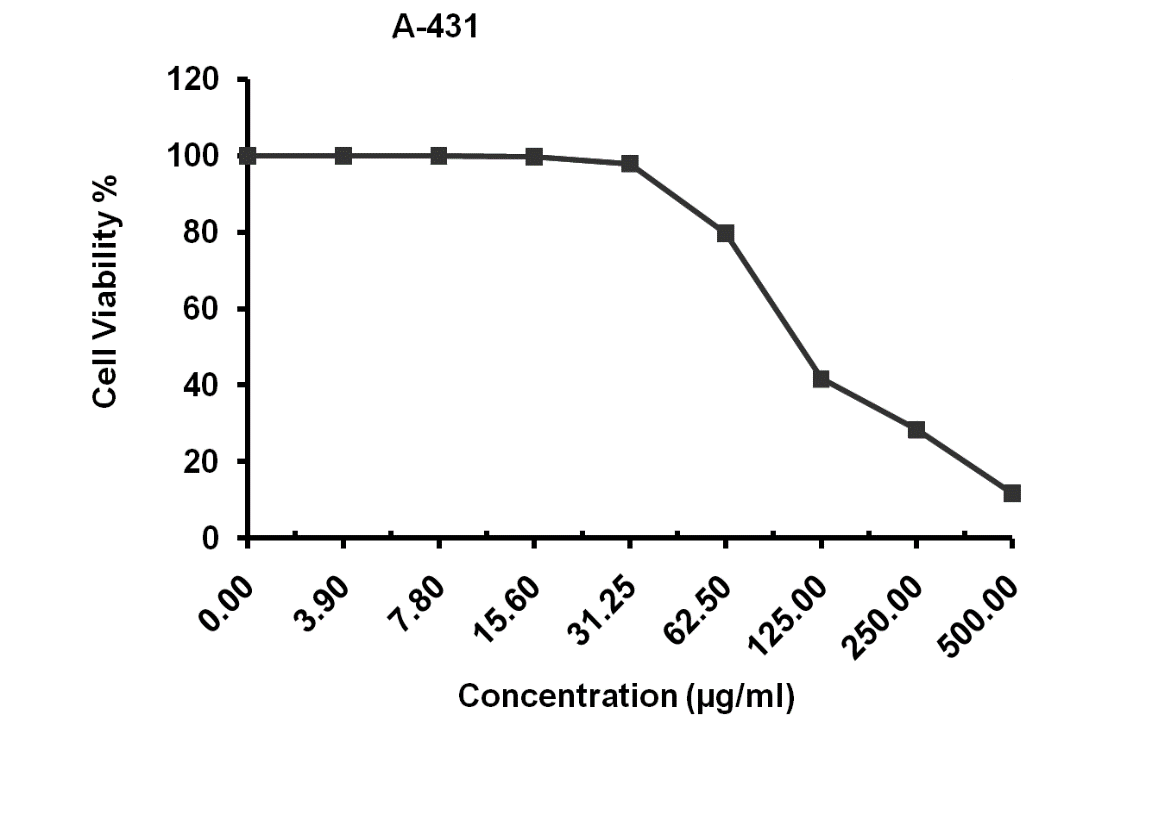


| **Sample conc. (µg/ml)** | **Viability%**  **(3 Replicates)** | | | **Mean** | **Inhibitory %** | **S.D. (±)** |
| --- | --- | --- | --- | --- | --- | --- |
|  | **1^st^** | **2^nd^** | **3^rd^** |  |  |  |
| **500** | **11.94** | **10.87** | **12.31** | **11.71** | **88.29** | **0.75** |
| **250** | **28.76** | **26.91** | **29.54** | **28.40** | **71.60** | **1.35** |
| **125** | **41.48** | **40.82** | **42.75** | **41.68** | **58.32** | **0.98** |
| **62.5** | **79.51** | **81.49** | **78.16** | **79.72** | **20.28** | **1.67** |
| **31.25** | **98.17** | **98.23** | **97.64** | **98.01** | **1.99** | **0.32** |
| **15.6** | **100** | **100** | **99.15** | **99.72** | **0.28** | **0.49** |
| **7.8** | **100** | **100** | **100** | **100.00** | **0.00** | **0.00** |
| **3.9** | **100** | **100** | **100** | **100.00** | **0.00** | **0.00** |
| **0** | **100** | **100** | **100** | **100** | **0** |  |

***Comment*:** The following **IC_50_ values** (presented as three readings) were detected against **skin cell line (A-431)** under the experimental conditions for 48 hrs.

| **Sample code** | **IC_50_ (µg/ml)**  **(3 Replicates)** | | | **Mean**  **IC_50_ (µg/ml)** | **S.D. (±)** |
| --- | --- | --- | --- | --- | --- |
|  | **1^st^** | **2^nd^** | **3^rd^** |  |  |
| **2** | **111.00** | **110.89** | **112.20** | **111.36** | **0.73** |

1. **Evaluation of cytotoxicity against MCF-7 cell line**


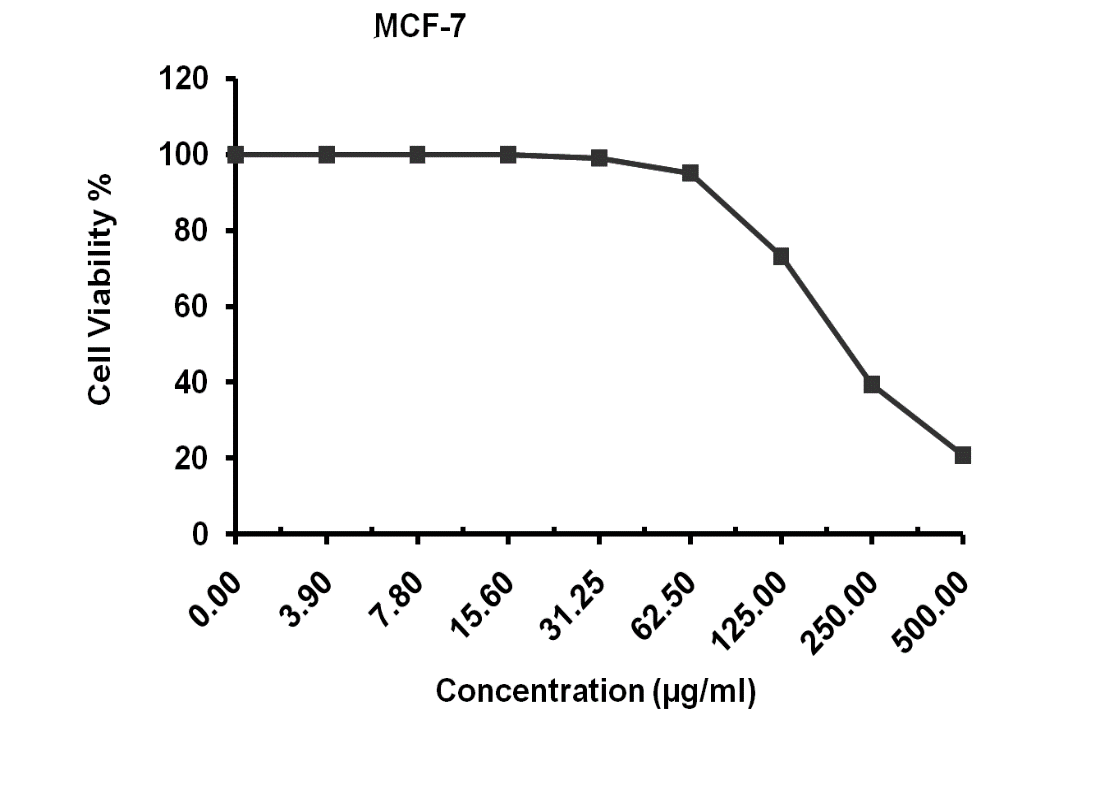


| **Sample conc. (µg/ml)** | **Viability%**  **(3 Replicates)** | | | **Mean** | **Inhibitory %** | **S.D. (±)** |
| --- | --- | --- | --- | --- | --- | --- |
|  | **1^st^** | **2^nd^** | **3^rd^** |  |  |  |
| **500** | **20.45** | **22.83** | **19.06** | **20.78** | **79.22** | **1.91** |
| **250** | **42.39** | **38.64** | **37.51** | **39.51** | **60.49** | **2.55** |
| **125** | **76.21** | **72.93** | **70.65** | **73.26** | **26.74** | **2.79** |
| **62.5** | **98.84** | **95.28** | **91.47** | **95.20** | **4.80** | **3.69** |
| **31.25** | **100** | **98.56** | **98.95** | **99.17** | **0.83** | **0.74** |
| **15.6** | **100** | **100** | **100** | **100.00** | **0.00** | **0.00** |
| **7.8** | **100** | **100** | **100** | **100.00** | **0.00** | **0.00** |
| **3.9** | **100** | **100** | **100** | **100.00** | **0.00** | **0.00** |
| **0** | **100** | **100** | **100** | **100** | **0** |  |

***Comment*:** The following **IC_50_ values** (presented as three readings) were detected against **breast cell line (MCF-7)** under the experimental conditions for 48 hrs.

| **Sample code** | **IC_50_ (µg/ml)**  **(3 Replicates)** | | | **Mean**  **IC_50_ (µg/ml)** | **S.D. (±)** |
| --- | --- | --- | --- | --- | --- |
|  | **1^st^** | **2^nd^** | **3^rd^** |  |  |
| **2** | **221.87** | **208.59** | **202.89** | **211.12** | **9.74** |

1. **Evaluation of cytotoxicity against MDA-MB-231 cell line**


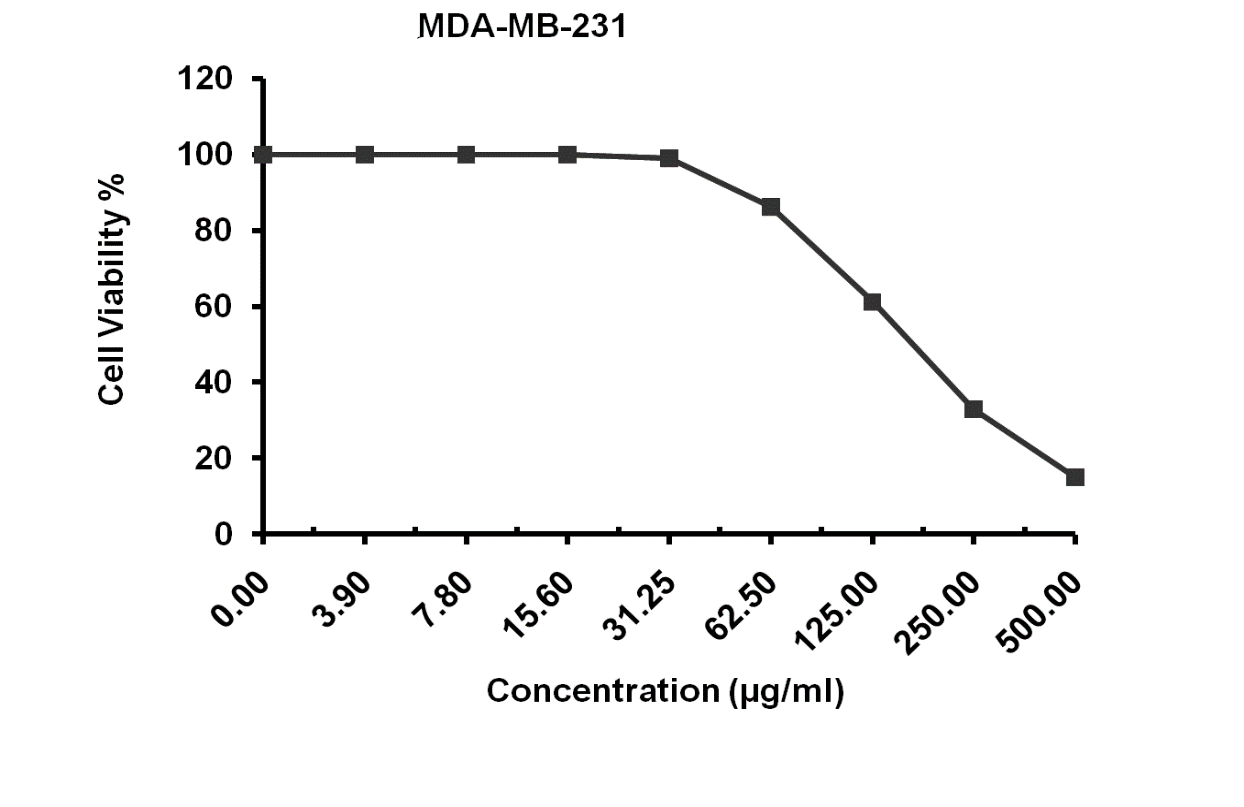


| **Sample conc. (µg/ml)** | **Viability%**  **(3 Replicates)** | | | **Mean** | **Inhibitory %** | **S.D. (±)** |
| --- | --- | --- | --- | --- | --- | --- |
|  | **1^st^** | **2^nd^** | **3^rd^** |  |  |  |
| **500** | **14.57** | **13.86** | **16.40** | **14.94** | **85.06** | **1.31** |
| **250** | **32.98** | **31.72** | **34.05** | **32.92** | **67.08** | **1.17** |
| **125** | **62.85** | **59.43** | **61.37** | **61.22** | **38.78** | **1.72** |
| **62.5** | **86.20** | **85.36** | **87.04** | **86.20** | **13.80** | **0.84** |
| **31.25** | **99.41** | **98.79** | **98.79** | **99.00** | **1.00** | **0.36** |
| **15.6** | **100** | **100** | **100** | **100.00** | **0.00** | **0.00** |
| **7.8** | **100** | **100** | **100** | **100.00** | **0.00** | **0.00** |
| **3.9** | **100** | **100** | **100** | **100.00** | **0.00** | **0.00** |
| **0** | **100** | **100** | **100** | **100** | **0** |  |

***Comment*:** The following **IC_50_ values** (presented as three readings) were detected against **breast cell line (MDA-MB-231)** under the experimental conditions for 48 hrs.

| **Sample code** | **IC_50_ (µg/ml)**  **(3 Replicates)** | | | **Mean**  **IC_50_ (µg/ml)** | **S.D. (±)** |
| --- | --- | --- | --- | --- | --- |
|  | **1^st^** | **2^nd^** | **3^rd^** |  |  |
| **2** | **178.77** | **167.54** | **177.02** | **174.45** | **6.05** |

1. **Evaluation of cytotoxicity against HepG-2 cell line**


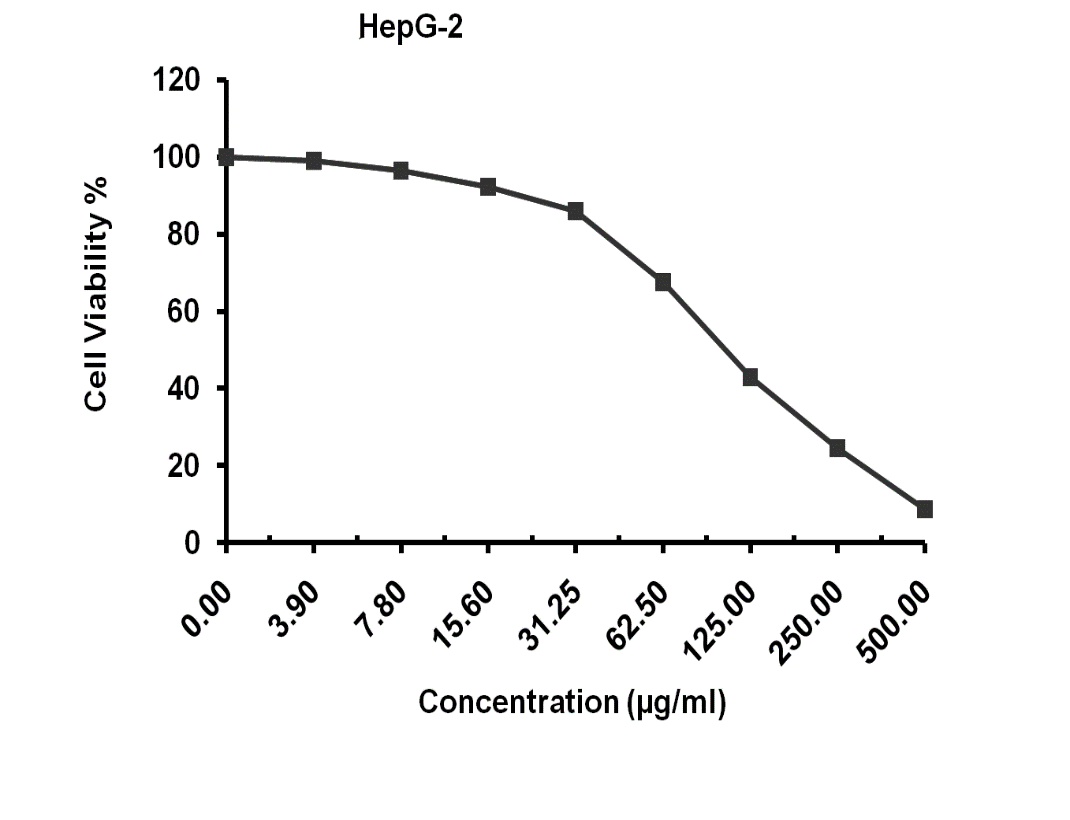


| **Sample conc. (µg/ml)** | **Viability%**  **(3 Replicates)** | | | **Mean** | **Inhibitory %** | **S.D. (±)** |
| --- | --- | --- | --- | --- | --- | --- |
|  | **1^st^** | **2^nd^** | **3^rd^** |  |  |  |
| **500** | **8.45** | **9.72** | **8.09** | **8.75** | **91.25** | **0.86** |
| **250** | **25.93** | **24.28** | **23.72** | **24.64** | **75.36** | **1.15** |
| **125** | **43.17** | **44.45** | **41.28** | **42.97** | **57.03** | **1.59** |
| **62.5** | **65.91** | **69.23** | **67.54** | **67.56** | **32.44** | **1.66** |
| **31.25** | **86.02** | **87.49** | **84.27** | **85.93** | **14.07** | **1.61** |
| **15.6** | **93.46** | **92.16** | **91.48** | **92.37** | **7.63** | **1.01** |
| **7.8** | **97.89** | **95.47** | **96.12** | **96.49** | **3.51** | **1.25** |
| **3.9** | **99.04** | **98.59** | **99.37** | **99.00** | **1.00** | **0.39** |
| **0** | **100** | **100** | **100** | **100** | **0** |  |

***Comment*:** The following **IC_50_ values** (presented as three readings) were detected against **Hepatocellular carcinoma cell line (HepG-2)** under the experimental conditions for 48 hrs.

| **Sample code** | **IC_50_ (µg/ml)**  **(3 Replicates)** | | | **Mean**  **IC_50_ (µg/ml)** | **S.D. (±)** |
| --- | --- | --- | --- | --- | --- |
|  | **1^st^** | **2^nd^** | **3^rd^** |  |  |
| **2** | **106.23** | **111.00** | **104.25** | **107.16** | **3.47** |

1. **Evaluation of cytotoxicity against HCT-116 cell line**


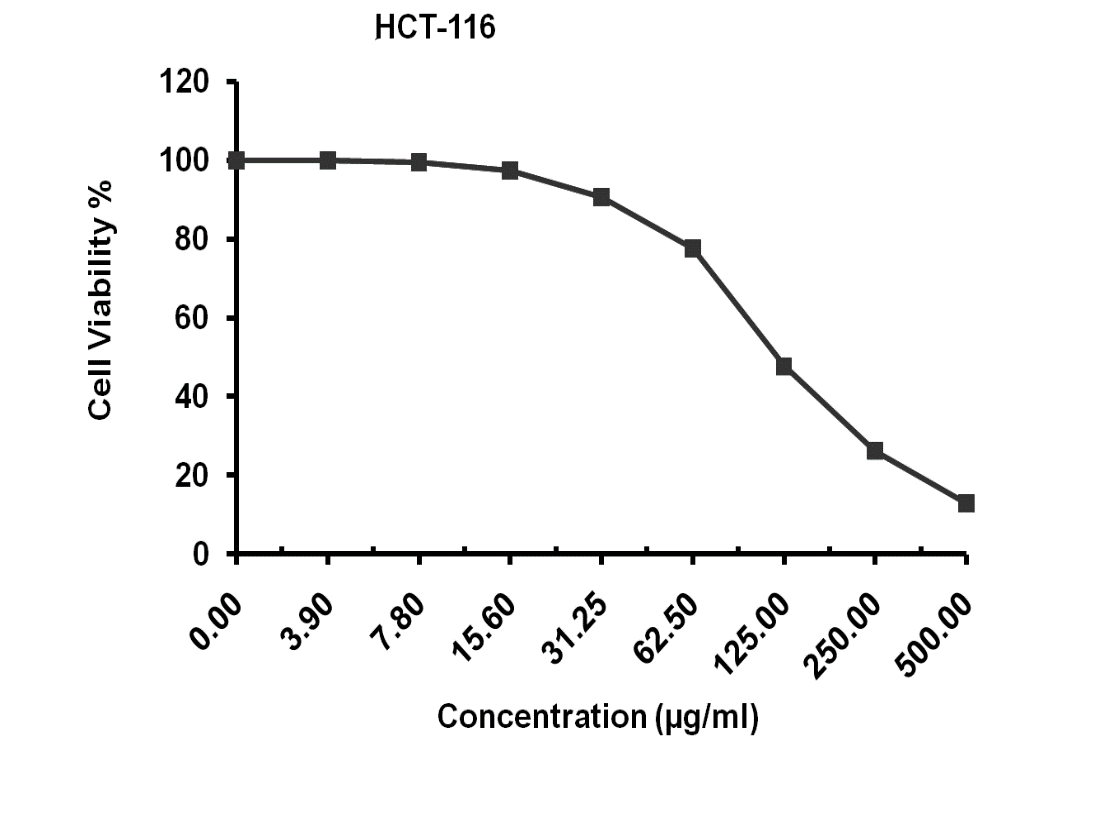


| **Sample conc. (µg/ml)** | **Viability%**  **(3 Replicates)** | | | **Mean** | **Inhibitory %** | **S.D. (±)** |
| --- | --- | --- | --- | --- | --- | --- |
|  | **1^st^** | **2^nd^** | **3^rd^** |  |  |  |
| **500** | **12.47** | **14.05** | **11.92** | **12.81** | **87.19** | **1.11** |
| **250** | **26.89** | **27.13** | **24.61** | **26.21** | **73.79** | **1.39** |
| **125** | **48.96** | **48.96** | **45.13** | **47.68** | **52.32** | **2.21** |
| **62.5** | **81.78** | **76.21** | **74.95** | **77.65** | **22.35** | **3.63** |
| **31.25** | **92.47** | **90.56** | **89.03** | **90.69** | **9.31** | **1.72** |
| **15.6** | **98.02** | **97.94** | **96.42** | **97.46** | **2.54** | **0.90** |
| **7.8** | **99.68** | **100** | **98.76** | **99.48** | **0.52** | **0.64** |
| **3.9** | **100** | **100** | **100** | **100** | **0** | **0** |
| **0** | **100** | **100** | **100** | **100** | **0** |  |

***Comment*:** The following **IC_50_ values** (presented as three readings) were detected against **colorectal cell line (HCT-116)** under the experimental conditions for 48 hrs.

| **Sample code** | **IC_50_ (µg/ml)**  **(3 Replicates)** | | | **Mean**  **IC_50_ (µg/ml)** | **S.D. (±)** |
| --- | --- | --- | --- | --- | --- |
|  | **1^st^** | **2^nd^** | **3^rd^** |  |  |
| **2** | **123.02** | **122.61** | **114.79** | **120.14** | **4.64** |
