# Supplementary material for: Phytochemical and biological assessment of secondary metabolites isolated from a rhizosphere strain, Sphingomonas sanguinis DM of Datura metel
Source: BMC Complement Med Ther. 2024 May 25;24:205. doi: 10.1186/s12906-024-04482-6 (PMC11128111; doi:10.1186/s12906-024-04482-6)
Supplement: Supplementary file 2 — Supplementary Material 2 [file 12906_2024_4482_MOESM2_ESM.docx]

**Phytochemical and Biological Assessment of Secondary Metabolites Isolated from a Rhizosphere Strain, *Sphingomonas sanguinis* DM of *Datura metel***

Mohamed A. Awad^1,2^

mohamed.abo-elfadl@ejust.edu.eg

Sherif F.Hammad^3,5^

sherif.hammad@ejust.edu.eg

Samir F. El-Mashtoly^1^

samir.elmashtoly@ejust.edu.eg

Bahig El-Deeb^2^

bahig1978@gmail.com

Hesham S. M. Soliman^4,5*^

[hesham.soliman@ejust.edu.eg](mailto:hesham.soliman@ejust.edu.eg)

^1^Biotechnology Program, Institute of Basic and Applied Science, Egypt-Japan University of Science and Technology (E-JUST), New Borg El-Arab City, Alexandria 21934, Egypt

^2^Botany and Microbiology Department, Faculty of Science, Sohag University, Sohag 82524, Egypt

^3^Department of Pharmaceutical Chemistry, Faculty of Pharmacy, Helwan University, Helwan, Cairo 11795, Egypt

^4^Department of Pharmacognosy, Faculty of Pharmacy, Helwan University, Helwan, Cairo 11795, Egypt

^5^PharmD Program, Egypt-Japan University of Science and Technology (E-JUST), New Borg El-Arab City, Alexandria 21934, Egypt

* Corresponding author: [hesham.soliman@ejust.edu.eg](mailto:hesham.soliman@ejust.edu.eg)

**Molecular Characterization of the Selected Bacterial Isolate (Alignment)**


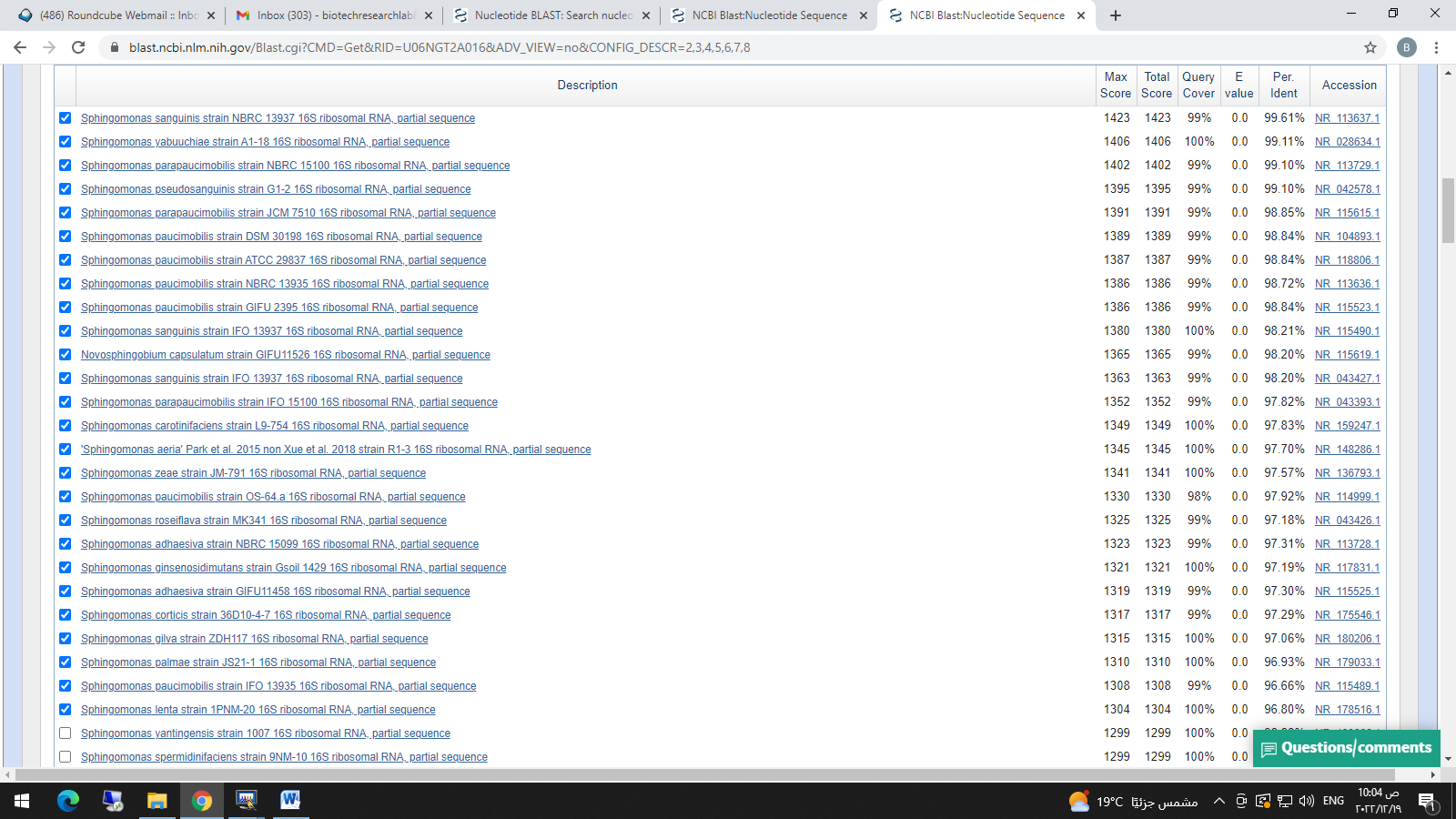


Query 3 AACGAACGCTGGCGGCATGCCTAACACATGCAAGTCGAACGAAGGCTTCGGCCTTAGTGG 62

||||||||||||||||||||||||||||||||||||||||||||||||||||||||||||

Sbjct 1 AACGAACGCTGGCGGCATGCCTAACACATGCAAGTCGAACGAAGGCTTCGGCCTTAGTGG 60

Query 63 CGCACGGGTGCGTAACGCGTGGGAATCTGCCCTTTGGTTCGGAATAACAGCTGGAAACGG 122

|||||||||||||||||||||||||||||||||| |||||||||||||||||||||||||

Sbjct 61 CGCACGGGTGCGTAACGCGTGGGAATCTGCCCTTAGGTTCGGAATAACAGCTGGAAACGG 120

Query 123 CTGCTAATACCGGATGATGACGAAAGTCCAAAGATTTATCGCCAGAGGATGAGCCCGCGT 182

||||||||||||||||||||||||||||||||||||||||||| ||||||||||||||||

Sbjct 121 CTGCTAATACCGGATGATGACGAAAGTCCAAAGATTTATCGCCTGAGGATGAGCCCGCGT 180

Query 183 TGGATTAGGTAGTTGGTGGGGTAAAGGCCTACCAAGCCGACGATCCATAGCTGGTCTGAG 242

||||||||||||||||||||||||||||||||||||||||||||||||||||||||||||

Sbjct 181 TGGATTAGGTAGTTGGTGGGGTAAAGGCCTACCAAGCCGACGATCCATAGCTGGTCTGAG 240

Query 243 AGGATGATCAGCCACACTGGGACTGAGACACGGCCCAGACTCCTACGGGAGGCAGCAGTG 302

||||||||||||||||||||||||||||||||||||||||||||||||||||||||||||

Sbjct 241 AGGATGATCAGCCACACTGGGACTGAGACACGGCCCAGACTCCTACGGGAGGCAGCAGTG 300

Query 303 GGGAATATTGGACAATGGGCGCAAGCCTGATCCAGCAATGCCGCGTGAGTGATGAAGGCC 362

||||||||||||||||||||| ||||||||||||||||||||||||||||||||||||||

Sbjct 301 GGGAATATTGGACAATGGGCGAAAGCCTGATCCAGCAATGCCGCGTGAGTGATGAAGGCC 360

Query 363 CTAGGGTTGTAAAGCTCTTTTACCCGGGAAGATAATGACTGTACCGGGAGAATAAGCCCC 422

||||||||||||||||||||||||||||||||||||||||||||||||||||||||||||

Sbjct 361 CTAGGGTTGTAAAGCTCTTTTACCCGGGAAGATAATGACTGTACCGGGAGAATAAGCCCC 420

Query 423 GGCTAACTCCGTGCCAGCAGCCGCGGTAATACGGAGGGGGCTAGCGTTGTTCGGAATTAC 482

||||||||||||||||||||||||||||||||||||||||||||||||||||||||||||

Sbjct 421 GGCTAACTCCGTGCCAGCAGCCGCGGTAATACGGAGGGGGCTAGCGTTGTTCGGAATTAC 480

Query 483 TGGGCGTAAAGCGCACGTAGGCGGCTTTGTAAGTCAGAGGTGAAAGCCTGGAGCTCAACT 542

||||||||||||||||||||||||||||||||||||||||||||||||||||||||||||

Sbjct 481 TGGGCGTAAAGCGCACGTAGGCGGCTTTGTAAGTCAGAGGTGAAAGCCTGGAGCTCAACT 540

Query 543 CCAGAACTGCCTTTGAGACTGCATCGCTTGAATCCAGGAGAGGTCAGTGGAATTCCGAGT 602

||||||||||||||||||||||||||||||||||||||||||||||||||||||||||||

Sbjct 541 CCAGAACTGCCTTTGAGACTGCATCGCTTGAATCCAGGAGAGGTCAGTGGAATTCCGAGT 600

Query 603 GTAGAGGTGAAATTCGTAGATATTCGGAAGAACACCAGTGGCGAAGGCGGCTGACTGGAC 662

||||||||||||||||||||||||||||||||||||||||||||||||||||||||||||

Sbjct 601 GTAGAGGTGAAATTCGTAGATATTCGGAAGAACACCAGTGGCGAAGGCGGCTGACTGGAC 660

Query 663 TGGTATTGACGCTGAGGTGCGAAAGCGTGGGGAGCAAACAGGATTAGATACCCTGGTAGT 722

||||||||||||||||||||||||||||||||||||||||||||||||||||||||||||

Sbjct 661 TGGTATTGACGCTGAGGTGCGAAAGCGTGGGGAGCAAACAGGATTAGATACCCTGGTAGT 720

Query 723 CCACGCCGTAAACGATGATAACTAGCTGTCCGGGCACTTGGTGCTTGGGTGGCGCAGCT 781

|||||||||||||||||||||||||||||||||||||||||||||||||||||||||||

Sbjct 721 CCACGCCGTAAACGATGATAACTAGCTGTCCGGGCACTTGGTGCTTGGGTGGCGCAGCT 779
