# Supplementary material for: Phytochemical and biological assessment of secondary metabolites isolated from a rhizosphere strain, Sphingomonas sanguinis DM of Datura metel
Source: BMC Complement Med Ther. 2024 May 25;24:205. doi: 10.1186/s12906-024-04482-6 (PMC11128111; doi:10.1186/s12906-024-04482-6)
Supplement: Supplementary file 4 — Supplementary Material 4 [file 12906_2024_4482_MOESM4_ESM.docx]

**Phytochemical and Biological Assessment of Secondary Metabolites Isolated from a Rhizosphere Strain, *Sphingomonas sanguinis* DM of *Datura metel***

Mohamed A. Awad^1,2^

mohamed.abo-elfadl@ejust.edu.eg

Sherif F.Hammad^3,5^

sherif.hammad@ejust.edu.eg

Samir F. El-Mashtoly^1^

samir.elmashtoly@ejust.edu.eg

Bahig El-Deeb^2^

bahig1978@gmail.com

Hesham S. M. Soliman^4,5*^

[hesham.soliman@ejust.edu.eg](mailto:hesham.soliman@ejust.edu.eg)

^1^Biotechnology Program, Institute of Basic and Applied Science, Egypt-Japan University of Science and Technology (E-JUST), New Borg El-Arab City, Alexandria 21934, Egypt

^2^Botany and Microbiology Department, Faculty of Science, Sohag University, Sohag 82524, Egypt

^3^Department of Pharmaceutical Chemistry, Faculty of Pharmacy, Helwan University, Helwan, Cairo 11795, Egypt

^4^Department of Pharmacognosy, Faculty of Pharmacy, Helwan University, Helwan, Cairo 11795, Egypt

^5^PharmD Program, Egypt-Japan University of Science and Technology (E-JUST), New Borg El-Arab City, Alexandria 21934, Egypt

* Corresponding author: [hesham.soliman@ejust.edu.eg](mailto:hesham.soliman@ejust.edu.eg)

**Characterization and Identification of Bioactive Compound 2 via Structure Elucidation**


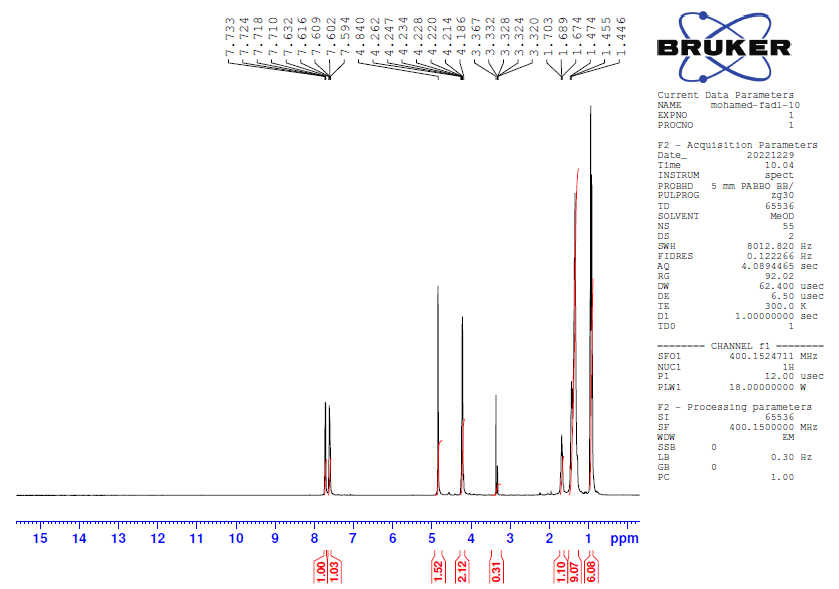


**Fig. S10** ^1^H NMR (400 MHz, MeOD) spectrum of compound **2**


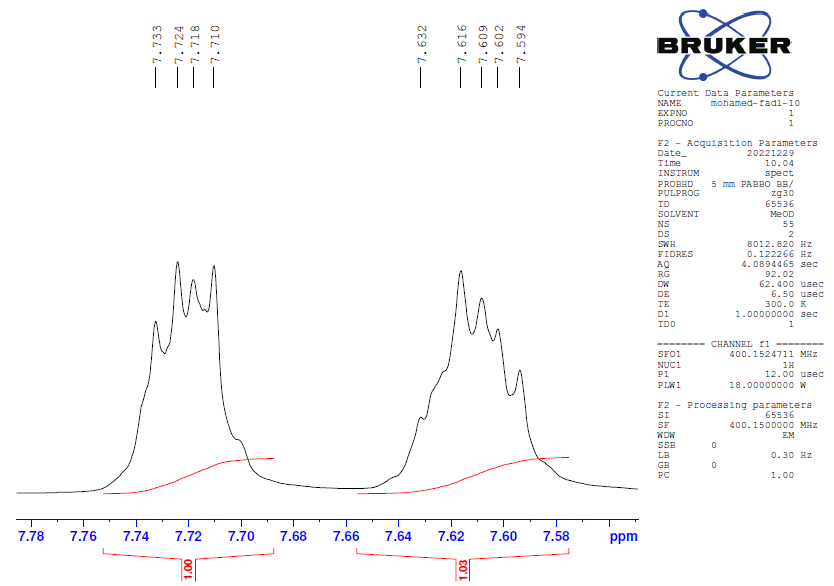


**Fig. S11** Magnification of ^1^H NMR (400 MHz, MeOD) spectrum of compound **2** (δ ppm 7.58 -7.78)


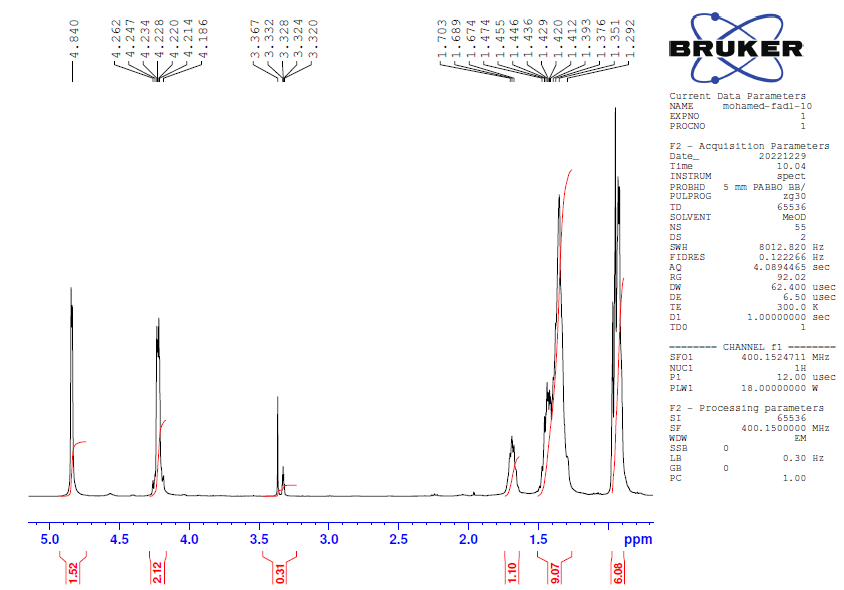


**Fig. S12** Magnification of ^1^H NMR (400 MHz, MeOD) spectrum of compound **2** (δ ppm 0.5 -5)


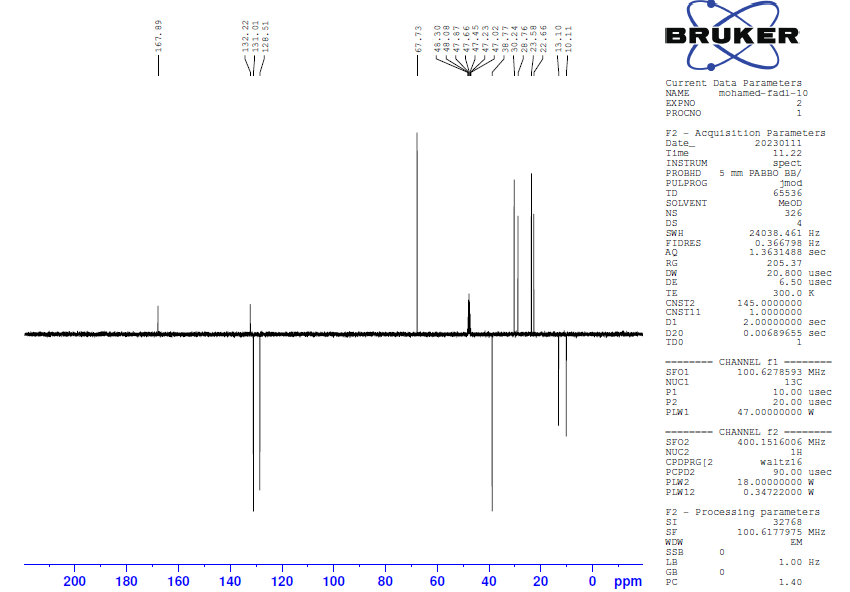


**Fig. S13** APT ^13^C NMR (100.63 MHz, MeOD), spectrum of compound **2**


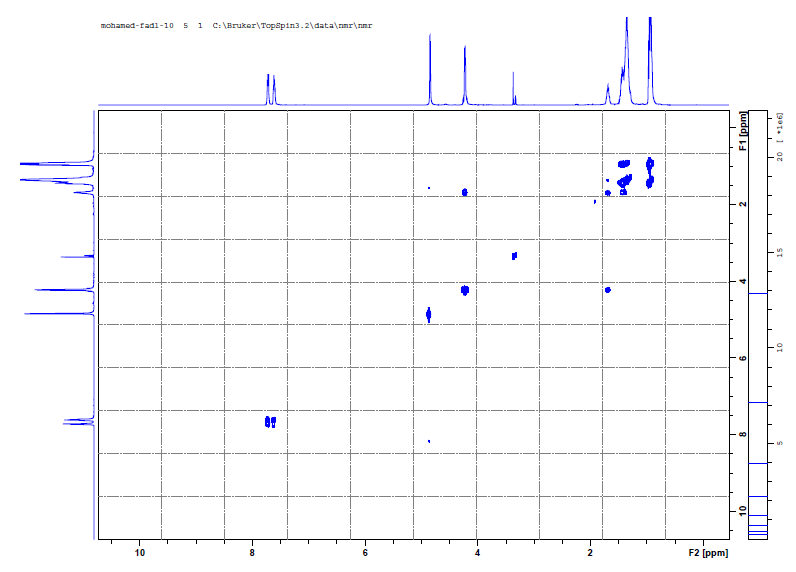


**Fig. S14** H-H COSY spectrum of compound **2**


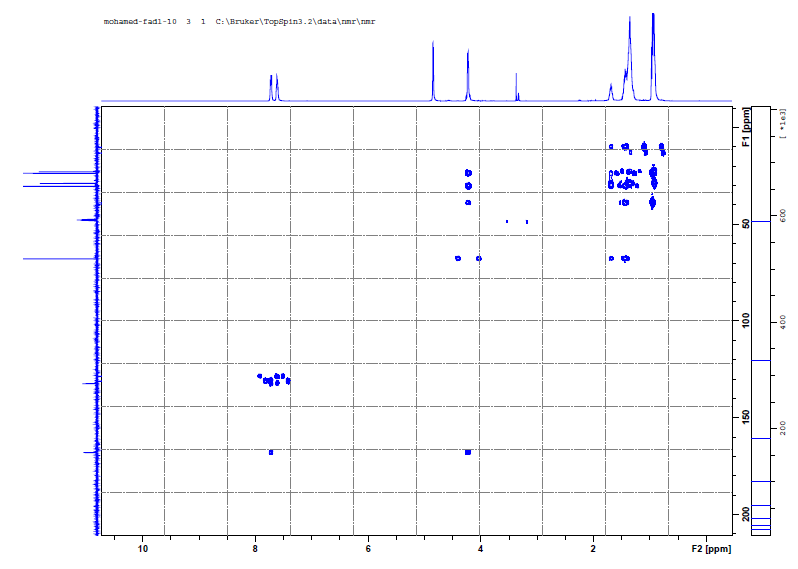


**Fig. S15** HMBC spectrum of compound **2**


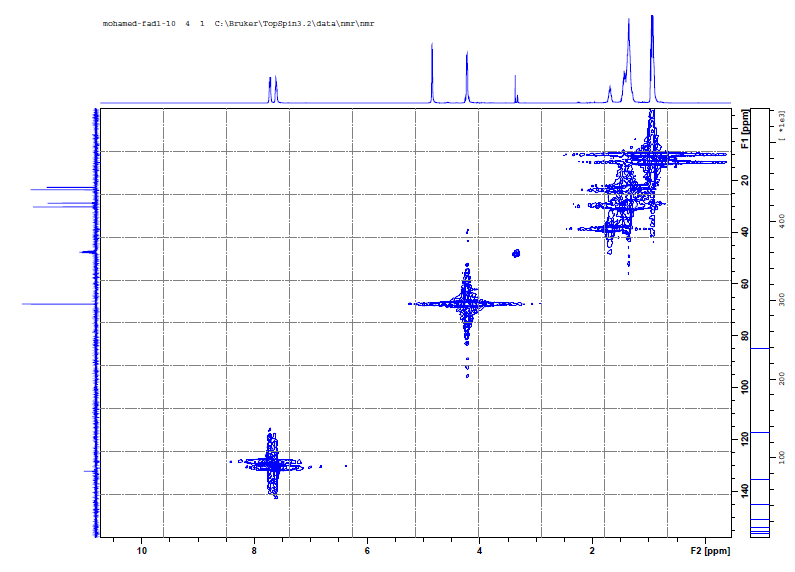


**Fig. S16** HMQC spectrum of compound **2**


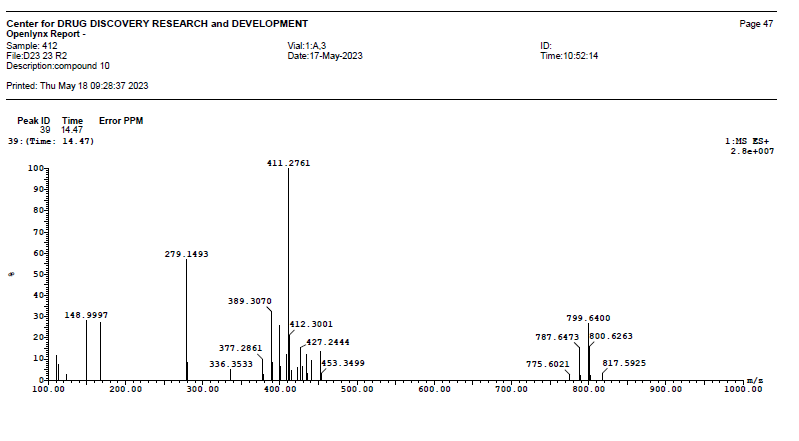


**Fig. S17** (+) ESI-MS spectrum of compound **2**

**Fig. S18** Absorbance of UV by compound **2**
